# Strain-Specific Gifsy-1 Prophage Genes Are Determinants for Expression of the RNA Repair Operon during the SOS Response in Salmonella enterica Serovar Typhimurium

**DOI:** 10.1128/jb.00262-22

**Published:** 2023-01-09

**Authors:** Jennifer E. Kurasz, Madison C. Crawford, Steffen Porwollik, Oliver Gregory, Katerina R. Tadlock, Eve C. Balding, Emily E. Weinert, Michael McClelland, Anna C. Karls

**Affiliations:** a Department of Microbiology, University of Georgia, Athens, Georgia, USA; b Department of Microbiology and Molecular Genetics, University of California–Irvine School of Medicine, Irvine, California, USA; c Department of Biochemistry and Molecular Biology, Pennsylvania State University, University Park, Pennsylvania, USA; University of California San Francisco

**Keywords:** *Salmonella*, *E. coli*, RpoN, sigma54, bacterial enhancer binding protein, bEBP activation, RtcR, SOS response, RecA, LexA, Gifsy prophages, RNA repair, transcription regulation, *Escherichia coli*, *Salmonella* Typhimurium

## Abstract

The adaptation of Salmonella enterica serovar Typhimurium to stress conditions involves expression of genes within the regulon of the alternative sigma factor RpoN (σ^54^). RpoN-dependent transcription requires an activated bacterial enhancer binding protein (bEBP) that hydrolyzes ATP to remodel the RpoN-holoenzyme-promoter complex for transcription initiation. The bEBP RtcR in *S.* Typhimurium strain 14028s is activated by genotoxic stress to direct RpoN-dependent expression of the RNA repair operon *rsr-yrlBA-rtcBA.* The molecular signal for RtcR activation is an oligoribonucleotide with a 3′-terminal 2′,3′-cyclic phosphate. We show in *S.* Typhimurium 14028s that the molecular signal is not a direct product of nucleic acid damage, but signal generation is dependent on a RecA-controlled SOS-response pathway, specifically, induction of prophage Gifsy-1. A genome-wide mutant screen and utilization of Gifsy prophage-cured strains indicated that the nucleoid-associated protein Fis and the Gifsy-1 prophage significantly impact RtcR activation. Directed-deletion analysis and genetic mapping by transduction demonstrated that a three-gene region (STM14_3218-3220) in Gifsy-1, which is variable between *S.* Typhimurium strains, is required for RtcR activation in strain 14028s and that the absence of STM14_3218-3220 in the Gifsy-1 prophages of *S. Typhimurium* strains LT2 and 4/74, which renders these strains unable to activate RtcR during genotoxic stress, can be rescued by complementation in *cis* by the region encompassing STM14_3218-3220. Thus, even though RtcR and the RNA repair operon are highly conserved in Salmonella enterica serovars, RtcR-dependent expression of the RNA repair operon in *S.* Typhimurium is controlled by a variable region of a prophage present in only some strains.

**IMPORTANCE** The transcriptional activator RtcR and the RNA repair proteins whose expression it regulates, RtcA and RtcB, are widely conserved in *Proteobacteria*. In Salmonella Typhimurium 14028s, genotoxic stress activates RtcR to direct RpoN-dependent expression of the *rsr-yrlBA-rtcBA* operon. This work identifies key elements of a RecA-dependent pathway that generates the signal for RtcR activation in strain 14028s. This signaling pathway requires the presence of a specific region within the prophage Gifsy-1, yet this region is absent in most other wild-type Salmonella strains. Thus, we show that the activity of a widely conserved regulatory protein can be controlled by prophages with narrow phylogenetic distributions. This work highlights an underappreciated phenomenon where bacterial physiological functions are altered due to genetic rearrangement of prophages.

## INTRODUCTION

**FIG 1:**
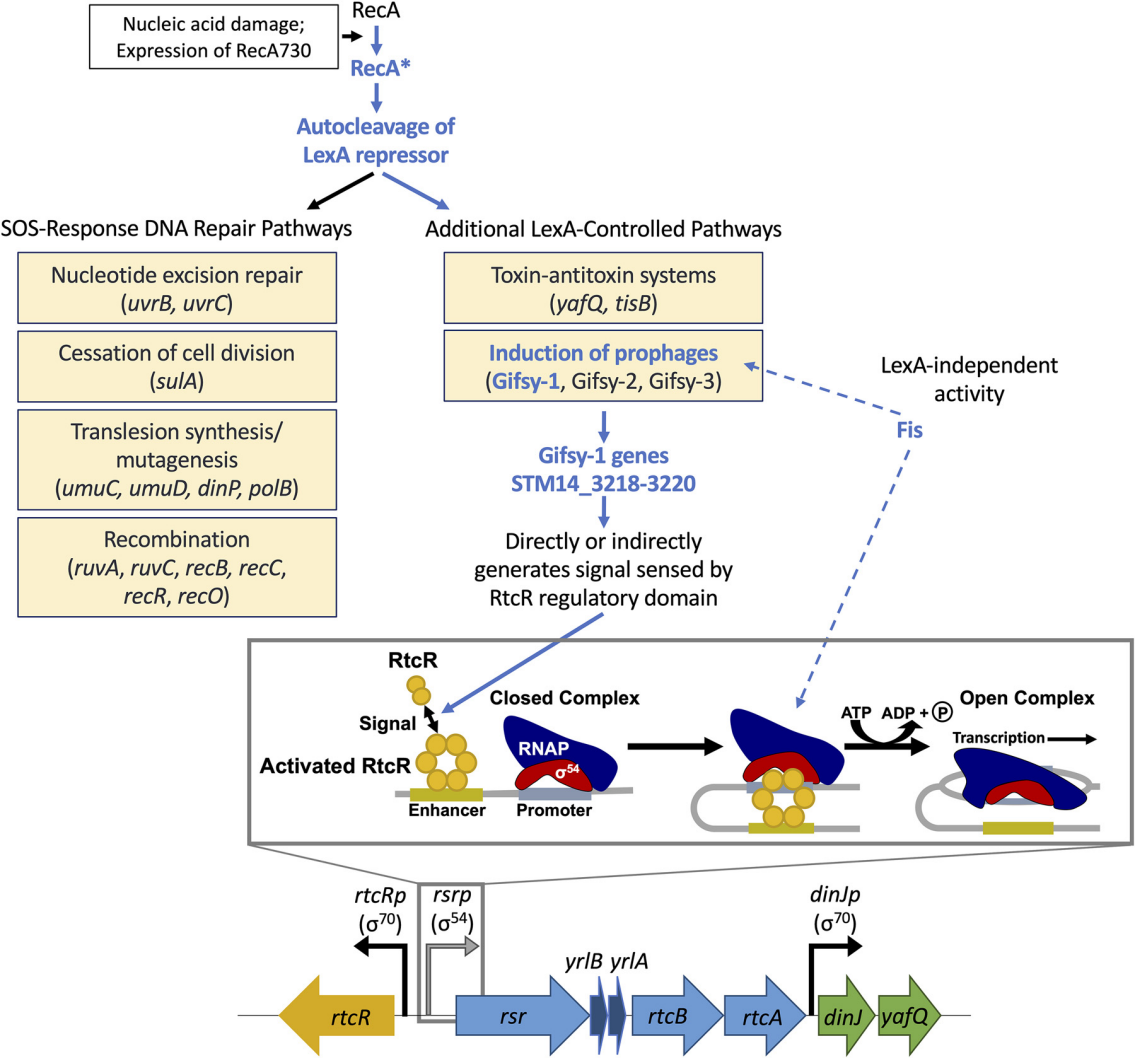
Regulatory circuit model for RecA-dependent RtcR activation of σ^54^-dependent transcription from *rsrp*. Activation of the bacterial enhancer-binding protein RtcR is required for σ^54^-dependent transcription from the *rsrp* promoter. *rsrp* controls transcription of the RNA repair operon, *rsr*-*yrlB*-*yrlA*-*rtcB*-*rtcA*, and the *dinJ-yafQ* type II toxin-antitoxin module, which is alternatively expressed from a σ^70^-type promoter (*dinJp*). *rtcR* is transcribed from a σ^70^-type promoter (*rtcRp*) oriented divergently from *rsrp*. A cellular signal binds to the regulatory domain of RtcR to activate the dimer to oligomerize into a complex that binds ATP and associates with an enhancer sequence upstream of the promoter. A DNA looping event brings the RtcR-enhancer complex into contact with the RNA polymerase holoenzyme (σ^54^-RNAP) bound to the promoter (closed complex). RtcR-mediated ATP hydrolysis drives the remodeling of σ^54^-RNAP to the open complex to initiate transcription. The RecA-dependent pathway that generates the cellular signal for RtcR activation is characterized in this study. Mutational analyses of RecA activation, LexA autocleavage, and LexA-controlled pathways (deleted genes/prophages that were assessed are indicated in parentheses) determined a pathway leading to induction of the Gifsy-1 prophage (essential pathway components are highlighted in blue). Strain-specific Gifsy-1 genetic determinants (STM14_3218-3220) and a bacterial gene product (Fis) are identified as essential components of the pathway; possible steps in the pathway for RtcR activation impacted by Fis are indicated by the dashed blue arrows.

The genome of the gastrointestinal pathogen Salmonella enterica serovar Typhimurium contains the *rsr-yrlBA-rtcBA* operon (Fig. 1), which is called the RNA repair operon based on the RNA splicing and repair functions of the metazoan and archaeal homologues of RtcB and RtcA (1–3). *In vitro* characterization of bacterial RtcB and RtcA confirmed RtcB as a noncanonical RNA ligase that joins 2′,3′-cyclic phosphate (2′,3′>PO_4_) or 3′-PO_4_ RNA ends with 5′-OH ends (2, 4) and RtcA as a terminal phosphate cyclase that generates the 2′,3′>PO_4_ ends (5, 6). While there is evidence that the target for RtcA and/or RtcB may be cleaved 16S rRNA in Escherichia coli (7–9) or tRNAs in *S.* Typhimurium strain 14028s (10), the physiological function of the RtcBA RNA repair system in bacteria is poorly understood. The first three genes in the *S.* Typhimurium RNA repair operon encode Rsr, YrlA, and YrlB, which are homologues of the metazoan Ro60 autoantigen and noncoding Y RNAs, respectively; Rsr and YrlA have been shown to bind exoribonuclease polynucleotide phosphorylase (PNPase), but an RNA substrate for this nucleoprotein complex has not been identified (11).

The *rsr-yrlBA-rtcBA* operon is under the transcriptional control of a σ^54^-dependent promoter, *rsrp* (12–14). σ^54^ (RpoN) is an alternative sigma factor that has been shown in *S.* Typhimurium strains LT2 and 14028s to direct RNA polymerase holoenzyme (Eσ^54^) to the promoters for genes whose products function in various environmental stress responses or utilization of alternative carbon sources (13, 14). Eσ^54^, unlike other Eσ, requires energy input in the form of ATP hydrolysis for open complex formation (reviewed in reference 15). The required ATPase activity is provided by bacterial enhancer binding proteins (bEBPs), a class of transcriptional regulators that typically consist of an N-terminal regulatory domain, a central AAA+ ATPase domain, and a C-terminal DNA binding domain that binds a specific enhancer site on the genome (reviewed in reference 16). bEBPs are generally activated by sensing a particular molecular signal through the regulatory domain; activation results in oligomerization, interaction with σ^54^, and hydrolysis of ATP for the energy-dependent remodeling of the Eσ^54^-promoter nucleoprotein complex (Fig. 1; *S.* Typhimurium bEBPs are reviewed in reference 17). RtcR is the primary bEBP responsible for regulating the σ^54^-dependent promoters for the *S.* Typhimurium 14028s and E. coli MG1655 RNA repair operons (7, 12). In *S.* Typhimurium 14028s, the RtcR regulon comprises the *rsr-yrlBA-rtcBA* operon and the downstream *dinJ-yafQ* toxin-antitoxin module (12). We previously demonstrated that RtcR-dependent expression of the RNA repair operon in *S.* Typhimurium 14028s is stimulated under nucleic acid-damaging conditions, e.g., treatment with mitomycin C (MMC), cisplatin, or hydrogen peroxide; also, deletion of *rtcR* or *rtcB* results in reduced cell viability following treatment with MMC (12).

Characterization of the molecular signal that activates RtcR and the cellular pathway that generates the signal will facilitate defining the role of the RNA repair system in Salmonella physiology and pathogenesis. Structural predictions for RtcR indicate that the regulatory domain is a divergent member of the CRISPR-associated Rossmann fold (CARF) domain superfamily and is likely to bind a modified nucleic acid (18, 19). We previously reported that expression of a mammalian 2′,3′-cyclic nucleotide 3′-phosphodiesterase in *S.* Typhimurium 14028s blocks activation of RtcR, suggesting that an important structural feature of the activating ligand is a terminal 2′,3′-cyclic phosphate (20). The YafQ endoribonuclease, encoded in the toxin-antitoxin module that is cotranscribed with the RNA repair operon in 14028s, yields RNA cleavage products with 2′,3′-cyclic phosphate ends; however, we demonstrated that YafQ is not required for RtcR activation (12). Recently, Hughes et al. showed that tRNA fragments accumulate during MMC treatment of *S.* Typhimurium 14028s and demonstrated that cleaved tRNA fragments with 2′,3′-cyclic phosphate ends bind and activate RtcR; however, the endoribonuclease and tRNA substrate that generate the signaling ligand under genotoxic conditions were not evident (10).

Our earlier studies showed that RtcR-dependent expression of the RNA repair operon in *S*. Typhimurium 14028s is dependent on RecA (12). RecA is the master regulator of the SOS response which controls DNA repair pathways, toxin-antitoxin systems, and prophage induction (reviewed in reference 21). RecA converts to its active form, RecA*, in the presence of single-stranded DNA (ssDNA) such as that found at stalled replication forks due to the presence of DNA lesions. RecA* acts as a coprotease for the LexA repressor, which controls expression of most genes responsible for DNA repair during the SOS response, and functions in other processes, including homologous recombination, cocleavage of repressors for some prophages (e.g., Fels and P22 in Salmonella strains), and regulation of the activities of SOS-induced polymerases Pol IV and Pol V. The essential link between RecA and RtcR activation provides a key to further defining the signal-generating pathway that leads to expression of the *S.* Typhimurium RNA repair operon. The steps in the RecA-controlled SOS response examined in our experimental approaches to defining the RtcR activation pathway are outlined in Fig. 1.

We show in this study that a constitutively active variant of RecA, RecA730, activates high levels of RtcR-dependent expression from *rsrp* in the absence of exogenous nucleic acid-damaging agents, indicating that the molecular signal is generated as a consequence of RecA activation, rather than from specific nucleic acid damage. An uncleavable variant of LexA prevents activation of RtcR; however, reporter assays for expression of the RNA repair operon with mutants defective for each of the LexA-controlled SOS-response DNA repair pathways indicate that none of these pathways are essential for RtcR activation. A transposon-based, genome-wide mutagenesis screen for genes that alter RecA-dependent RtcR activation revealed that the Gifsy-1 and Gifsy-3 prophages of *S.* Typhimurium strain 14028s, which are induced to their lytic state during SOS-activating conditions, play a role in activating RtcR. In assays with strains of 14028s that are cured for individual, or a combination of, Gifsy prophages, the Gifsy-1 prophage is shown to be essential for RtcR activation. Strikingly, strain-specific differences prevent expression of the RNA repair operon in *S.* Typhimurium strains LT2, 4/74, and SL1344 during the SOS response; the essential genomic differences impacting RtcR activation were mapped to a three-gene region, STM14_3218-3220, within the immunity region of Gifsy-1, which exhibits significant variability between Salmonella strains. The Tn*5* insertion mutant screen also identified the nucleoid protein Fis as playing an important role in RtcR-dependent transcription from the RNA repair operon promoter. This study provides significant and unexpected insights into the regulation of the RNA repair operon, which will assist in defining its physiological function.

## RESULTS

### A RecA*-controlled process generates the signal for RtcR activation in *S.* Typhimurium but not E. coli.

We previously found that RtcR-dependent expression of the RNA repair operon in *S*. Typhimurium 14028s occurs following nucleic acid damage that induces a strong SOS response, including treatment with MMC, cisplatin, or high levels of H_2_O_2_ (12). Under these genotoxic conditions, expression of the RNA repair operon is dependent on RecA, as little to no expression was detected in a Δ*recA* strain (12).

To address whether the signal that activates RtcR is a direct product of the nucleic acid-damaging agent or is generated by a RecA-dependent cellular process associated with the response to DNA damage, a plasmid was constructed that expresses a variant of RecA based on the *recA730* allele identified in E. coli (22). RecA730 contains a single amino acid substitution (E29K) that abolishes the requirement for RecBCD- or RecFOR-mediated loading onto ssDNA, leading to constitutively active RecA*. We introduced an equivalent mutation into the *S.* Typhimurium *recA* gene by overlap extension PCR to create the RecA* expression vector pJK15 and confirmed constitutive activity of the *S.* Typhimurium RecA730 (see Fig. S1 in the supplemental material). Heterologous expression of RecA730 in the absence of DNA-damaging conditions resulted in RecA* activity which relieved LexA-mediated repression of *sulA*, assessed by cell filamentation (Fig. S1A), and *recN*, measured by beta-galactosidase expression from a *recN*::MudJ transcriptional fusion (Fig. S1B). Under the same conditions, cells expressing wild-type RecA (RecA_WT_) did not exhibit RecA* activity (Fig. S1). In addition to confirming that *S.* Typhimurium RecA730 behaves in a manner consistent with what was reported for E. coli, these experiments show that *recA730* is a dominant allele when *recA*_WT_ is present on the chromosome, negating the need to work exclusively in a *recA* mutant strain.

**FIG 2:**
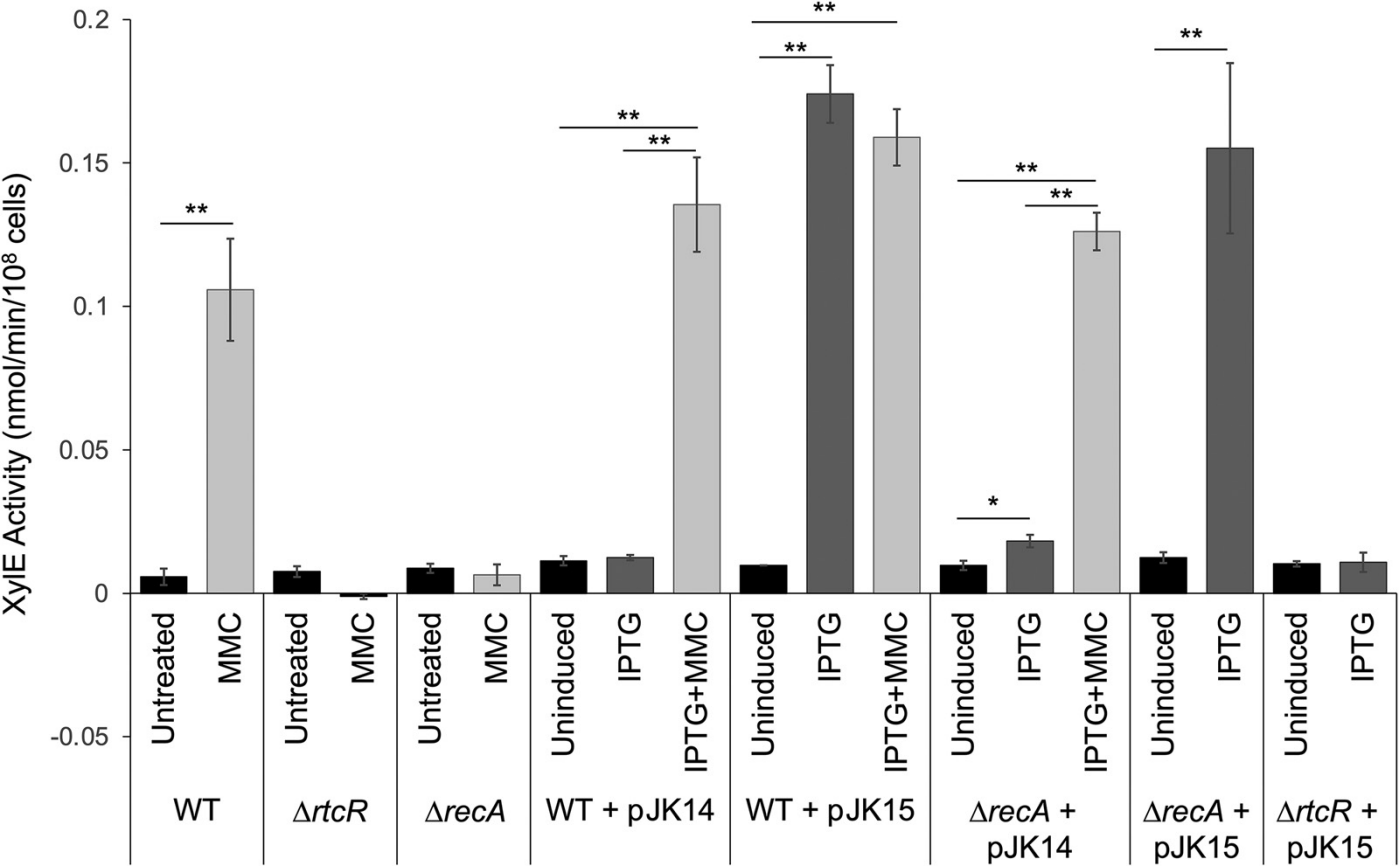
RecA730 induces RtcR-dependent activation of *rsrp* in the absence of DNA damage. XylE activity assays were conducted with WT (JEK17, Δ*rsr*::*xylE*), Δ*recA* (JEK26, Δ*recA*::*kan* Δ*rsr*::*xylE*), and Δ*rtcR* (JEK41, Δ*rtcR*::*kan* Δ*rsr*::*xylE*) reporter strains to assess expression from the *rsr* promoter during SOS response-inducing conditions, i.e., treatment with 3 μM MMC and/or 1 mM IPTG to induce expression of the constitutively active RecA730 variant from pJK15. WT RecA was expressed from pJK14 (1 mM IPTG induction). All data shown are representative of at least 3 biological replicates, each with two technical replicates; error bars represent ±1 standard deviation. Significant differences in XylE activity between treated samples and untreated samples are indicated (*, *P* < 0.05; **, *P* < 0.01; the ends of the horizontal bars are centered above the two compared data sets).

Expression of the RNA repair operon from *rsrp* in the presence of RecA730 was assessed using a previously constructed reporter strain in which *rsr*, the first gene of the operon, was replaced on the chromosome with *xylE*, which encodes catechol-2,3-dioxygenase (12). Promoter activation, quantified as the level of XylE activity, was compared to controls that were treated with 3 μM MMC, which results in the highest level of expression determined for *rsrp* under genotoxic conditions (12). Consistent with previous results, XylE activity was 18.4-fold upregulated in wild-type (WT) cells after a 90-min treatment with MMC, while increased XylE activity was not observed in a Δ*rtcR* or Δ*recA* strain (Fig. 2). In cells heterologously expressing RecA_WT_ (from plasmid pJK14), increased XylE activity was only observed when cells were concurrently treated with MMC to induce the SOS response (10.9-fold upregulated compared to cells expressing RecA_WT_ with no MMC), indicating that heterologous expression of RecA is not sufficient to stimulate promoter activity (Fig. 2). However, when RecA730 was expressed (from plasmid pJK15), XylE activity was nearly identical to levels seen in the MMC-treated control (17.9-fold upregulated compared to uninduced cells); activity did not increase further with concurrent MMC treatment (Fig. 2). A Δ*recA* strain behaved similarly to the WT strain when complemented with RecA_WT_ or RecA730 (Fig. 2). Importantly, no increase in XylE activity was detected in the Δ*rtcR* strain expressing RecA730, confirming that expression of the RNA repair operon in response to RecA730 is through activation of RtcR to simulate transcription from *rsrp* (Fig. 2). This indicates that the RtcR-activating signal is being generated in cells expressing RecA730 and is not dependent on a product from nucleic acid damage.

We considered whether RecA may not be responsible for signal generation but, rather, may be important for some other aspect of RtcR functionality, such as binding enhancer sites or interacting with the RpoN-holoenzyme. To address this, we expressed a constitutively-active RtcR variant (RtcR_con_) in a Δ*recA* Δ*rsr*::*xylE* reporter strain. RtcR_con_ lacks its N-terminal regulatory domain and does not require a molecular signal for activation, leading to constitutive stimulation of RpoN-dependent transcription from *rsrp* in the absence of nucleic acid damage (12). RtcR_con_ expression in the Δ*recA* strain resulted in high levels of XylE activity (Fig. S2), similar to what is observed in a WT strain (12), suggesting that RecA is not required for RtcR functionality after it is activated by the binding of a molecular signal to the regulatory domain. Thus, it is most likely that RecA is involved in generating the signaling ligand.

We previously found that genotoxic conditions that activate the RNA repair operon in *S.* Typhimurium, such as MMC treatment, do not activate the analogous *rtcBA* operon in E. coli MG1655, even though E. coli RtcR can functionally complement an *S.* Typhimurium Δ*rtcR* mutant and the promoter for the E. coli *rtcBA* operon is activated by RtcR_con_ (12). Since MMC is a well-known inducer of the SOS response in E. coli (23), this suggests that RecA* is not universally sufficient for initiating a signaling pathway for RtcR activation in species that encode the bEBP and RNA repair genes. To explore this further, we expressed the same RecA730 variant in an E. coli reporter strain in which *rtcB* was replaced with *xylE* on the chromosome. Induction of the SOS response in E. coli by the *S.* Typhimurium RecA730 variant was confirmed by observation of cell filamentation (Fig. S3A). However, in reporter assays, no significant activity was detected from the *rtcBA* promoter during RecA730 induction (Fig. S3B). Thus, RecA* activity in E. coli MG1655 does not appear to regulate the *rtcBA* operon. This result is consistent with recent work by Hughes et al. showing that, unlike in *S.* Typhimurium 14028s, treatment of E. coli MG1655 with MMC does not result in accumulation of cleaved tRNA fragments, the predicted ligand for RtcR activation in *S.* Typhimurium 14028s (10). However, earlier work by Engl et al. indicated that RtcR of E. coli MG1655 can be activated in the presence of tRNA fragments with 2′,3′-cyclic phosphate ends generated by ectopic expression of *S.* Typhimurium LT2 VapC or exogenous treatment with colicin D (7), suggesting that similar signals for RtcR activation are generated in E. coli and *S.* Typhimurium through different stress-response pathways.

### LexA cleavage is essential for RtcR activation, but not through induction of SOS repair pathways.

**FIG 3:**
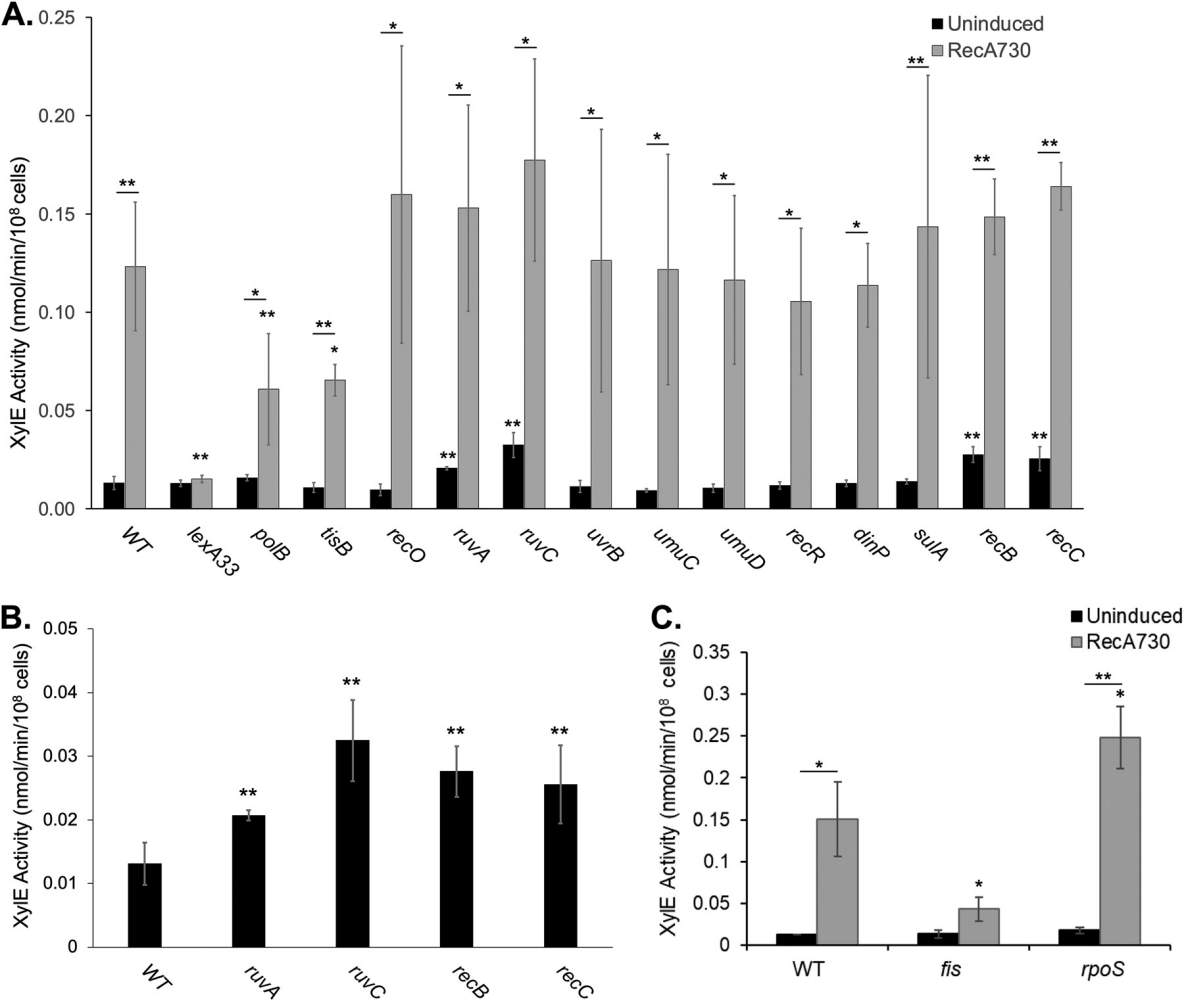
RtcR-dependent activation of the RNA repair operon in mutants that target SOS response pathways and functions identified as involved in RtcR activation in a genome-wide mutagenesis screen. (A) XylE activity assays were conducted on single-gene deletion mutants for genes that encode components of the SOS response (see Fig. 1). The designated gene was replaced with a *kan* resistance marker, with the exception of *lexA33*, which has *lexA* replaced with a *cam* resistance marker fused to the *lexA*3 allele that encodes a noncleavable variant of LexA. All strains carry pJK15 (RecA730), and cells were assayed after growth without inducing agent or with 1 mM IPTG. (B) Deletion mutants in panel A that showed enhanced activation of the *rsr* promoter in the absence of the inducing agent; note the change in scale of the *y* axis. (C) Bacterial genes identified in the Tn*5* mutagenesis screen with significant impacts on RtcR-dependent activation. All data shown are representative of at least 3 biological replicates, each with two technical replicates; error bars represent ±1 standard deviation. Significant differences in XylE activity between uninduced and induced (RecA730 expression) samples are indicated by asterisks above horizontal bars centered between the paired samples; significance differences in XylE activity for mutant strains and treatments (uninduced or induced) in comparison to the WT counterpart are indicated by asterisks above the mutant samples (*, *P* < 0.05; **, *P* < 0.01).

In order to determine whether the involvement of RecA in RtcR activation is through its interactions with the LexA repressor, we assayed for transcription from *rsrp* in a *lexA33* mutant, which generates uncleavable LexA and therefore prohibits the expression of SOS response genes. We found that induction of RecA730 expression in the *lexA33* mutant did not result in RtcR-dependent transcription from *rsrp* (Fig. 3A; Table 1). Therefore, RecA-mediated generation of the signal for RtcR activation requires expression of one or more genes that are directly regulated by LexA.

**TABLE 1:** Identified *S.* Typhimurium genes that alter expression from the RtcR-dependent promoter

Gene(s) targeted		Fold-change XylE activity for:^*c*^	
		Δ mutant vs WT uninduced	Δ mutant vs WT induced
SOS response			
*recA*^*a*^		1.51	–16.4 (**)
*lexA*		–1.01	–8.33 (**)
*ruvA*		1.58 (**)	1.24
*ruvC*		2.48 (**)	1.44
*recB*		2.10 (**)	1.21
*recC*		1.95 (**)	1.33
*polB*		1.21	–2.04 (**)
*tisB*		–1.20	–1.89 (*)
Prophages^*b*^			
Gifsy-1 prophage		–1.25	–19.2 (**)
STM14_3218-3220		1.30	–50.0 (**)
Gifsy-3 prophage		1.80 (**)	–1.85 (*)
Gifsy-1, -2 prophages		–1.18	–116 (**)
Gifsy-1, -2, -3 prophages		–1.75 (**)	–116 (**)
Other Genes			
*fis*		1.04	–3.45 (*)
*rpoS*		1.37	1.65 (*)

a Induction for the *recA* strain was with 1 μg/mL MMC. All other strains were induced by RecA730.

b Gifsy-2 is not listed because deletion of this prophage did not affect RtcR activation (Fig. 4A).

c Asterisks denote the statistical significance (two-sample *t* test) of the fold change in XylE activity for a particular mutant strain and treatment (uninduced or induced) compared to the WT counterpart (*, *P* < 0.05; **, *P* < 0.01).

We previously assayed differential gene expression for MMC-treated and untreated WT *S.* Typhimurium 14028s by transcriptome sequencing (RNA-seq); 22 LexA-regulated genes were identified as upregulated >3-fold in cells treated with MMC (12). These upregulated genes overlap known SOS response genes in E. coli that are involved in nucleotide excision repair, homologous recombination (HR), mutagenic translesion synthesis, cessation of cell division, and toxin-antitoxin systems. Given the requirement for cleavable LexA, we hypothesized that the RtcR-activating signal may be generated through one of the LexA-regulated repair pathways (see Fig. 1). To test this, we generated mutants of the *xylE* reporter strain with deletions in individual genes that are essential to specific repair pathways: *uvrB* and *uvrC* of the UvrABC nucleotide excision repair system, Pol IV (*dinP*) and DNA polymerase II (*polB*) for mostly error-free translesion DNA synthesis, Pol V subunits (*umuC* and *umuD*) for mutagenic translesion DNA synthesis, Holliday junction DNA helicase (*ruvA*) and crossover junction endodeoxyribonuclease (*ruvC*) for resolution of HR intermediates, *recO* and *recR* as components of the RecFOR HR pathway, *recB* and *recC* as components of the RecBCD HR pathway, and *sulA* for cessation of cell division while DNA lesions are repaired during the SOS response. We additionally tested a deletion mutant for the LexA-regulated gene *tisB* (*ysdB*), which encodes an inner membrane-targeting toxin (24) and was the most highly upregulated SOS response gene in the RNA-seq data set (12).

These single-gene deletion mutants were assayed for XylE activity with and without induction of the SOS response by heterologously expressed RecA730 (Fig. 3A). In the absence of RecA730, the RNA repair operon is 1.6- to 2.5-fold upregulated in *ruvA*, *ruvC*, *recB*, and *recC* mutants compared to the WT strain (Fig. 3B; Table 1). These results are consistent with the low-level chronic expression of the SOS response seen in *ruvA* or *ruvC* mutants of E. coli (25, 26) and with identification of *ruvA*, *ruvC*, and *recA* in a genome-wide Tn*5* insertion screen for mutations that activate expression from *rsrp* in *S.* Typhimurium 14028s (10). Under inducing conditions for expression of RecA730, XylE activity in the Δ*tisB* and Δ*polB* strains was 1.88-fold and 2.07-fold reduced, respectively, compared to the WT induced strain. While the decreases in XylE activity in the *tisB* and *polB* mutants relative to the WT strain were statistically significant, the low level of change suggests a small, indirect, or partly redundant role for TisB or PolB in generating the signal for RtcR activation. XylE activity in each of the other deletion mutants for the SOS response DNA repair pathways did not differ significantly from that of the WT (Fig. 3A; Table 1). The persistence of expression from the RNA repair operon promoter in each of the strains that are defective for one of the SOS DNA repair pathways suggests that either these pathways do not play an essential role in RtcR activation or there are additional genes within the genome that can compensate for the specific deletions that we examined. For instance, there is some redundancy in the activities of mutagenic DNA polymerases such as Pol IV and Pol V. The roles of other pathways controlled by RecA*-LexA in *S.* Typhimurium, i.e., lysogeny/lytic pathways of Salmonella prophages, are addressed below.

### Genome-wide screen for *S.* Typhimurium genes involved in RtcR activation.

To more broadly assess cellular functions required for RtcR activation, we designed a genome-wide screen utilizing a library of barcoded Tn*5* insertion mutants (27). P22 phage was used to transduce DNA from a Tn*5* insertion mutant library of *S.* Typhimurium 14028s (~40,000 independent insertion mutants) into WT 14028s containing the RecA730 expression vector (pJK15) and a single-copy reporter plasmid with the RNA repair operon promoter, *rsrp* with an upstream regulatory sequence, fused to the *lacZY* operon (pJK19). On medium containing IPTG (isopropyl-β-d-thiogalactopyranoside) and X-Gal (5-bromo-4-chloro-3-indolyl-β-d-galactopyranoside), WT colonies appeared light blue, reflecting the modest level of LacZ expression from the tightly regulated σ^54^ promoter upon activation of the SOS response. Screening of 73,000 individual colonies identified 101 putative downregulated mutants (white colonies) and 35 upregulated mutants (dark blue colonies) that were confirmed via a secondary screen for the relevant phenotype. Each group of mutants was pooled, and the barcoded regions of the Tn*5* elements were sequenced. Locations of the Tn*5* insertions were identified based on an existing map of the barcoded Tn*5* elements to the genome (27). To eliminate noise that might arise due to mutants that had multiple Tn*5* insertions from transduction with more than one phage particle, the following criteria were applied for inclusion in the list of Tn*5* insertions identified for downregulated and upregulated mutants (Table S1): (i) multiple insertions mapped to the same gene, (ii) multiple insertions mapped to genes within the same operon, or (iii) insertions mapped to multiple genes that are functionally linked.

Tn*5* insertions affecting both bacterial and prophage genes were identified in the downregulated and upregulated mutant pools (Table S1); prophage-specific mutations are addressed separately below. Among the Tn*5* insertions that downregulated expression from *rsrp*, multiple insertions were identified within *rtcR*, confirming that mutations affecting RtcR-dependent activation of transcription from *rsrp* could be identified from this screen. To confirm a role in RtcR activation for other genes associated with Tn*5* insertions in the downregulated or upregulated mutants, we examined the activity of *rsrp* in 14028s reporter strains with the gene of interest deleted. Single-gene deletion mutants were generated in WT 14028s containing the *rsrp-lacZ* fusion reporter plasmid (pJK19) and/or in the chromosomal reporter strain JEK17 (Δ*rsr*::*xylE*); each reporter strain additionally contained pJK15 (RecA730 expression plasmid) to activate expression from *rsrp*. In beta-galactosidase assays, the Δ*fis*::*kan* and Δ*pcnB*::*kan* mutants exhibited a significant difference in LacZ activity compared to the WT control under inducing conditions (Fig. S4A). However, for the *xylE* reporter strains, only the Δ*fis* mutant consistently showed a significant decrease in XylE activity relative to the WT control (Fig. 3C; Fig. S4B; Table 1). Importantly, when comparing the activity of the *fis* mutant under inducing versus noninducing conditions, there was no significant increase in either LacZ or XylE activity, suggesting that Fis plays an essential role in the RtcR signaling pathway and/or *rsrp* activation (Fig. 3C; Fig. S4A; Table 1). Consistent with the potential key role for Fis in the activation of RtcR and/or transcription from *rsrp*, XylE activity for the WT 14028s Δ*rsr*::*xylE* reporter strain shows a dependence on growth phase (Fig. S5) that correlates with the peak of *fis* transcript/Fis protein levels seen in E. coli at the early log phase following outgrowth of stationary-phase cells in rich medium and the decrease to undetectable levels by the early stationary phase (28, 29).

From the Tn*5* insertions that upregulated LacZ expression in the colony screen, the associated bacterial genes *hupA*, *hupB*, and *rpoS* were assessed in the reporter assays; only the Δ*rpoS* mutant exhibited statistically significant enhanced activation of the RNA repair operon promoter upon expression of RecA730 compared to the WT (Fig. 3C; Fig. S4; Table 1), but the increase was less than 2-fold. This small increase in RtcR-activated expression from *rsrp* in the *rpoS* mutant may simply reflect a loss of competition by the alternative sigma factor RpoS for core RNA polymerase, allowing formation of more RpoN holoenzyme to interact with activated RtcR for transcription from the RpoN-dependent *rsrp* (30).

Some of the Tn*5* insertions identified in the plate screen did not have a confirmed impact on *rsrp* activation in reporter assays with mutants containing the corresponding gene deletions (Fig. S4). Perhaps the different growth conditions for the Tn*5* screen (48 h on solid medium) and reporter assays (aerobic cultures at mid-log phase) altered cell physiology, which is very likely to affect cellular pathways leading to activation of RtcR and RpoN-dependent transcription of the RNA repair operon. Another plausible explanation for some of the mutant phenotypes is that the Tn*5* insertion may have conferred polar or position-specific effects in the colony screen, which were not reproduced when the gene was fully deleted.

### Prophages play a role in RtcR activation in *S.* Typhimurium strain 14028s.

In addition to the bacterial repair pathways of the SOS response, RecA*-LexA control the induction of multiple lambdoid phages within the *S.* Typhimurium genome (31). Strain 14028s, in which the Tn*5* insertion mutant analysis was conducted, has three active prophages (Gifsy-1, Gifsy-2, and Gifsy-3) and a defective ST64B prophage. Other strains of *S.* Typhimurium carry different combinations of prophages: LT2 carries Gifsy-1 and Gifsy-2 but not Gifsy-3 or ST64B and contains additional prophages, Fels-1 and Fels-2, while strains 4/74 and SL1344 (which is a derivative of 4/74) carry Gifsy-1, Gifsy-2, and ST64B but neither of the Fels phages or Gifsy-3 (Salmonella prophages are reviewed in reference 32). Several of these prophages contain genes that are important for Salmonella pathogenicity and, like other prophages, their lytic genes are repressed during bacterial growth by a series of transcriptional repressors and early terminators. The Gifsy phages are regulated by repressors that do not undergo proteolysis; rather, lytic genes are expressed when the repressor associates with a cognate antirepressor, whose gene is transcriptionally controlled by LexA (33). Induction of the SOS response signals the lysogenized phages to convert to their lytic state (32).

In our genome-wide Tn*5* mutagenesis screen, many of the mutations that caused differential expression from the RNA repair operon promoter were insertions within Gifsy-1 or Gifsy-3, but no mutations were identified within Gifsy-2 (Table S1). In Gifsy-1, insertions in STM14_3218 and STM14_3220, which are located in the immunity region, resulted in downregulation of *rsrp*. STM14_3218 is likely a member of the AAA family ATPases and has a weak homology with the 401-amino acid ParA of the virulence plasmid in the same strain (58 amino acid identities in a region of 216 amino acids; 27%). Proteins of the ParA family play a role in plasmid or chromosome partitioning; however, ParA homologues with this function require the interaction of ParB, for which there is no homologous gene within Gifsy-1. STM14_3220 is annotated as a hypothetical protein of unknown function. In Gifsy-3, Tn*5* insertions in genes within the recombination region resulted in enhanced expression from *rsrp*, including insertions in the genes encoding integrase (*int*), excisionase (*xis*), RecT, exodeoxyribonuclease VIII (*exo*), and RecE. An insertion mutation within a single gene of unknown function in this same region, as well as an insertion in a lysozyme-encoding gene, both resulted in downregulation of *rsrp*. These results suggested that both Gifsy-1 and Gifsy-3 may contribute to the RtcR activation pathway.

To further investigate the *S.* Typhimurium prophages involved in generating the molecular signal to activate RtcR, we constructed 14028s Δ*rsr*::*xylE* reporter strains that were cured of one or more of the Gifsy prophages. RecA730 was expressed as the activating condition for transcription of the RNA repair operon, and XylE levels were compared to those of the equivalent WT reporter strain (Fig. 4A). When 14028s was cured of all three Gifsy prophages, the strain was no longer able to express XylE from the RNA repair operon promoter. Although Tn*5* insertions in the replication region of Gifsy-3 led to enhanced activation of the RNA repair operon promoter in the genome-wide screen (Table S1), a strain that was cured for only Gifsy-3 showed modestly lower XylE activity under activation conditions compared to WT (1.9-fold; Fig. 4A and Table 1). Strains cured of Gifsy-1 and Gifsy-2, or only Gifsy-1, showed no increase in XylE activity when RecA730 expression was induced compared to an uninduced control (Fig. 4A), which is consistent with the phenotype of mutants with Tn*5* insertions in Gifsy-1 genes STM14_3218 and STM14_3220 (Table S1). The strain cured of only Gifsy-2 did not exhibit altered XylE expression from *rsrp* (Fig. 4A). These results show that Gifsy-1 plays an essential role in the RtcR signaling network in strain 14028s, with additional contributions by Gifsy-3.

**FIG 4:**
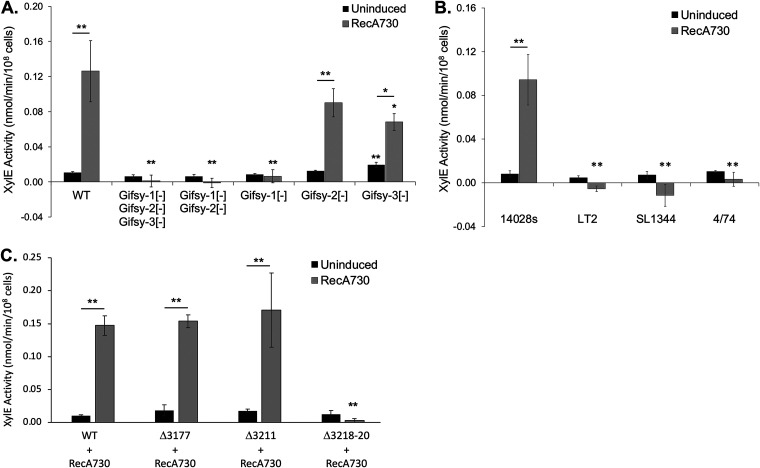
RtcR-dependent expression from the *rsr* promoter is dependent on prophage determinants. (A) XylE assays were conducted on *S.* Typhimurium 14028s Δ*rsr*::*xylE* reporter strains that were cured of single or multiple prophages: JEK87 (WT), JEK60 (Gifsy-1[-] Gifsy-2[-] Gifsy-3[-]), JEK86 (Gifsy-1[-] Gifsy-2[-]), JEK83 (Gifsy-1[-]), JEK84 (Gifsy-2[-]), and JEK85 (Gifsy-3[-]). All strains carried IPTG-inducible pJK15 (RecA730 expression plasmid), and cells were assayed after growth without an inducing agent or with 1 mM IPTG (90 min). (B) XylE assays were conducted on different strains of *S.* Typhimurium with the Δ*rsr*::*xylE* reporter for expression of the RNA repair operon. All strains carried pJK15 (RecA730), and assays were conducted after growth without an inducing agent or with 1 mM IPTG (90 min). (C) XylE assays were performed with the 14028s WT, ΔSTM14_3177 (tail protein gene), ΔSTM14_3211 (potential endoribonuclease gene), and ΔSTM14_3218-3220 reporter strains which carried pJK15 (RecA730). XylE assays were conducted after growth without an inducing agent or induced with 1 mM IPTG (90 min). Significant differences in XylE activity between uninduced and induced samples are indicated by asterisks above horizontal bars centered between the paired samples; significance differences in XylE activity for mutant or different strains and treatments (uninduced or induced) in comparison to the 14028s WT counterpart are indicated by asterisks above the mutant or different strain samples (*, *P* < 0.05; **, *P* < 0.01). All data shown are representative of at least 3 biological replicates, each with two technical replicates; error bars represent ±1 standard deviation.

### Strain-specific genetic features of Gifsy-1 are required for RtcR activation in *S*. Typhimurium.

To assess the activation of RtcR in other Salmonella strains that naturally carry Gifsy-1 within their genome, we generated equivalent reporter strains of LT2, SL1344, and 4/74 by replacing *rsr* on the chromosome with *xylE*. Unexpectedly, the wild-type LT2, SL1344, and 4/74 reporter strains did not exhibit increased XylE activity when RecA730 was expressed (Fig. 4B), despite the presence of the apparently essential Gifsy-1. RpoN-dependent expression of the RNA repair operon in strains LT2 and SL1344 has previously been demonstrated using a constitutively-active bEBP variant (10, 13); thus, the likely cause for the lack of XylE activity in these strains is an inability to generate the molecular signal to activate wild-type RtcR .

**FIG 5:**
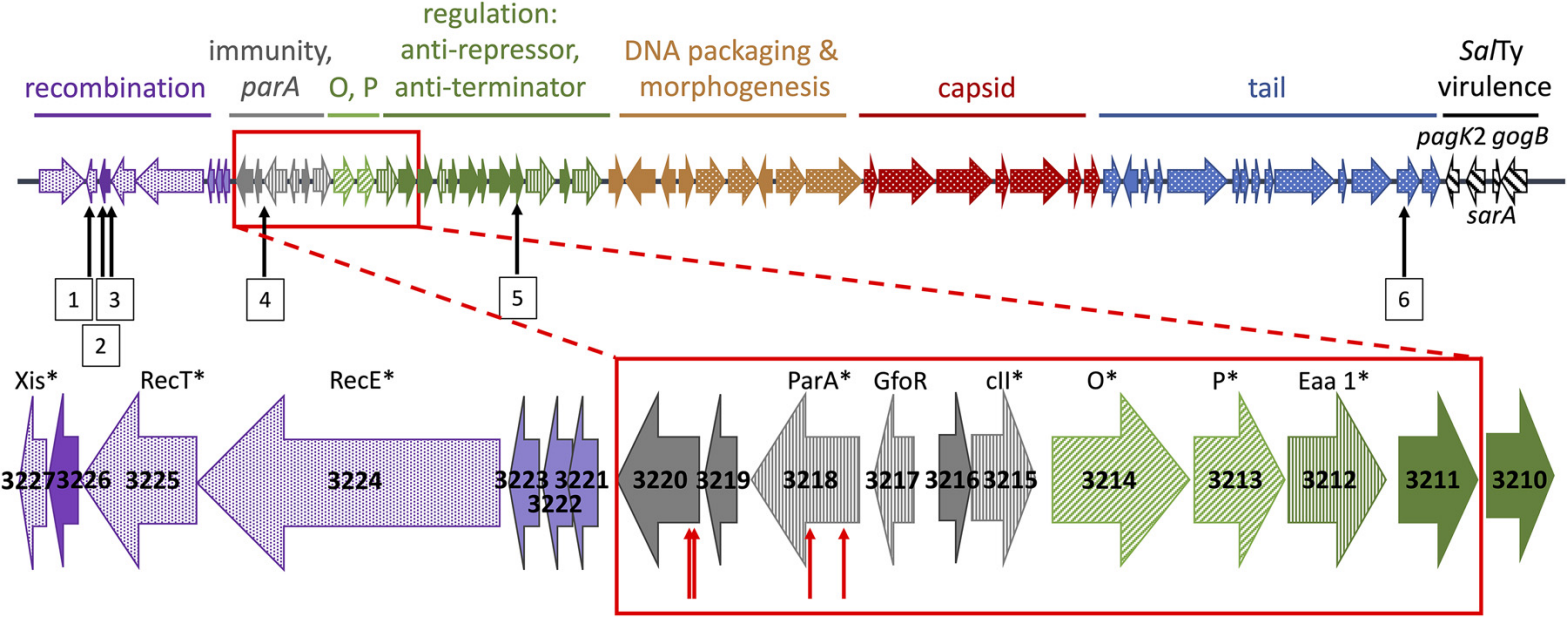
*S.* Typhimurium 14028s Gifsy-1 prophage map. The prophage map for Gifsy-1 is not drawn to scale. Genes are indicated by arrows; arrow color indicates the potential role in the phage lysogenic/lytic cycles, and solid-fill versus pattern-fill indicates whether the gene encodes a hypothetical protein or protein of known/predicted function, respectively. The 6.59-kb region of 14028s Gifsy-1 that exhibits a high degree of sequence dissimilarity with Gifsy-1 prophages in LT2 and SL1344 (4/74) is highlighted (expanded red box); the STM14 gene ID numbers are shown, and the asterisked gene products are based on sequence similarity with known phage/bacterial genes. Although it is unknown whether STM14_3217 to STM14_3220 are expressed as an operon, coregulation of these genes under induction conditions (MMC treatment) is suggested by previously reported RNA-seq analysis of differential gene expression in strain 14028s untreated versus MMC-treated which showed the level of expression of genes STM14_3217 through STM14_3220 was not significantly changed by MMC treatment, while expression of genes immediately upstream (STM14_3212 to STM14_3216) and downstream (STM14_3221 to STM14_3228) increased over 32-fold after MMC treatment (12). Upward red arrows indicate the position of independent insertions in STM14_3218 and STM14_3220 that resulted in downregulation of RtcR activation in the Tn*5* mutant library screen (Table S1); all four EZ-Tn5<Kan2>insertions are oriented antisense to the target gene. No insertion in STM14_3219 was identified in the screen, but the Tn*5* insertion mutant library had only one insertion in this 309-bp gene, while the library contained 223 and 103 insertions in STM14_3218 (840 bp) and STM14_3220 (663 bp), respectively. Upward black arrows indicate the positions of KanR cassettes associated with SGD_3227, SGD_3226, Chimera_1, Chimera_2, SGD_3240, and SGD_3169 (labeled 1 to 6, respectively) within the Gifsy-1 prophage of donor 14028s strains used in cotransduction assays with 4/74 and LT2 to map the RtcR-activation locus (Table S2).

It has previously been reported that there can be significant variation within the same prophage genome in different strains of Salmonella (33, 34); thus, we hypothesized that strain-specific differences within the Gifsy-1 genome contribute to activation of the RNA repair operon in 14028s, but not LT2, 4/74, or SL1344. Comparative analysis of the Gifsy-1 genomes specific to strains 14028s, LT2, and SL1344 (4/74) using NCBI BLAST highlighted a 6.59-kb region of the Gifsy-1_14028s_ genome that is dissimilar to the other two strains, spanning the genes designated STM14_3211-3220 (Fig. 5). This region includes STM14_3218 and STM14_3220 identified in our Tn*5* mutant screen (Table S1), an *eaa* homologue, genes for phage replication proteins P and O, a *cII*-like gene, the phage repressor gene *gfoR* (33), and three genes encoding hypothetical proteins. Structural prediction models for the hypothetical proteins encoded by STM14_3211, STM14_3219, and STM14_3220 were generated using Phyre2 software (35), and motif analysis was conducted using the Kyoto Encyclopedia of Genes and Genomes (KEGG) database (36); although no predicted function could be ascribed to the products of STM14_3219 or STM14_3220, the hypothetical protein encoded by STM14_3211 was predicted to contain a higher eukaryotes and prokaryotes nucleotide-binding (HEPN) domain. The HEPN superfamily consists of a large number of ribonucleases, toxins, restriction-modification enzymes, CRISPR-Cas proteins, and proteins involved in abortive infection, and in most of these, the HEPN domain functions as a metal-independent endoribonuclease (37). Since the RtcR-activating signal is likely a cleaved tRNA with 2′,3′-cyclic phosphate termini, such as those generated by metal-independent endoribonucleases, we considered STM14_3211, along with the STM14_3218-3220 region, to be good candidates for the strain-specific determinant of Gifsy-1_14028s_ that is essential for activating the RNA repair operon.

To address the RecA*-induced Gifsy-1_14028s_ determinant for RtcR activation, 14028s *xylE* reporter strains were generated with deletions of STM14_3211, STM14_3218-3220, or STM14_3177 (control mutant; a phage tail gene outside the strain-specific variable region). These reporter strains were transformed with the IPTG-inducible RecA730-expression plasmid and utilized in XylE assays (Fig. 4C). Deletion of STM14_3211 or STM14_3177 did not alter RtcR-activated expression of XylE from *rsrp*. However, deletion of STM14_3218-3220 significantly reduced expression of XylE from *rsrp* under activation conditions relative to the WT strain (50-fold decrease; Fig. 4C and Table 1), suggesting that a function(s) encoded by a gene(s) in the STM14_3218-3220 region is required for RtcR activation in the reporter strain. Insertions in STM14_3218 and STM14_3220 downregulated RtcR activation in the EZ-Tn*5*<KanR> insertion mutant screen, possibly indicating that both genes are required for RtcR activation; however, the anti-sense orientation of the insertions and potential coregulation of STM14_3218-3220 (see Fig. 5) suggest that the insertions in STM14_3218 may be polar on STM14_3219-3220.

A possible mechanism whereby deletion of STM14_3218-3220 may affect the readout of the reporter is destabilization of the plasmid used in the reporter system. To test this possibility, MMC was used to initiate activation conditions in the XylE assay, instead of expression of RecA730 from pJK15. The ΔSTM14_3218-3220 reporter strain still exhibited significantly reduced expression of XylE relative to the WT strain under activation conditions (13-fold; Fig. S6). This result demonstrates that the observed phenotype for deletion of STM14_3218-3220 in Gifsy-1 is not due to an effect specifically of RecA730 versus WT RecA nor an effect of the STM14_3218-3220 region on the copy number or segregation of the RecA730-expression plasmid.

To assess whether heterologous expression of STM14_3218-3220 could be utilized to further define the function(s) from this region required for RtcR activation, complementation of the ΔSTM14_3218-3220 reporter strain by STM14_3218-3220 expression from the arabinose-inducible *araBADp* promoter on pBAD30 (pJK21) was measured in the presence and absence of the IPTG-inducible RecA730-expression plasmid (pJK15). Expression of STM14_3218-3220 from pJK21 in the absence of RecA730 was not sufficient to activate expression of XylE, while expression of STM14_3218-3220 and RecA730 from pJK21 and pJK15, respectively, resulted in 15-fold higher XylE activity compared to that of the uninduced control (Fig. S7A). However, the level of XylE activity in the ΔSTM14_3218-3220 reporter strain expressing STM14_3218-3220 and RecA730 was 5-fold lower than that of the control WT reporter strain containing the empty pBAD30 expression vector and pJK15 induced with IPTG (Fig. S7A). We found that addition of arabinose to the WT control strain reduced XylE expression by 3-fold (Fig. S7A). Because the inducer for the expression vector had a negative effect on the WT reporter system, complementation assays in the ΔSTM14_3218-3220 reporter strain were then performed with STM14_3218-3220, STM14_3218, or STM14_3219-3220 under the control of the IPTG-inducible *lac* promoter on pSRK-Tc, and MMC treatment was used to induce activation conditions. No significant increase in XylE activity was observed in the ΔSTM14_3218-3220 reporter strain following MMC treatment and IPTG-induced heterologous expression of STM14_3218-3220, STM_3218, or STM14_3219-3220 (Fig. S7B). The results of these complementation assays may indicate that the required genetic determinant (e.g., DNA feature, small RNA (sRNA), protein product) for RtcR activation that is associated with the STM14_3218-3220 region of Gifsy-1 functions optimally in *cis*. The experiments described below demonstrate the transfer of the phenotype when the region is kept in *cis.*

### The *S*. Typhimurium 14028s strain-specific genetic determinants for RtcR-dependent expression of the RNA repair operon map to the variable region of prophage Gifsy-1.

For a more global approach to determining all genetic factors that account for the strain-specific differences in RtcR activation, we developed a screen for horizontal transfer of the *S.* Typhimurium strain 14028s genomic determinants for activation of RtcR to strain 4/74, which does not exhibit RtcR activation in response to RecA*. Using P22 transduction, ~41-kb segments of the 14028s genome were transferred from the Tn*5* insertion library in 14028s into strain 4/74 carrying pJK15 (expressing RecA730) and a reporter plasmid, pP*_rsr_-lacZ*, which has the full *lacZ* gene transcriptionally fused to the *rsr* promoter. The KanR-cassette associated with Tn*5* insertions allowed selection for chimeric 4/74-14028s transductants. Over 100,000 colonies were screened for *lacZ* expression from *rsrp*; blue colonies, which were potential 4/74 transductants that acquired 14028s genomic DNA encoding the factor(s) required for RtcR activations, arose at a frequency of ~0.002. Whole-genome sequencing was performed for four independent transductants that were confirmed to exhibit RecA*-dependent *lacZ* expression from *rsrp;* these 4/74-14028s chimeric strains are designated Chimera_1, _2, _3, and _4, respectively. All four chimeras have a deletion in the 4/74 genome extending approximately from position 2763600 to 2772500, within Gifsy-1_4/74_, and have replaced this region with a 14028s genomic DNA region that maps approximately from 2816000 to 2826000 in that genome (Fig. S8). This transduced region from 14028s encompasses the 6.59-kb region of variability identified in Gifsy-1_14028s_ compared to Gifsy-1 of LT2 and SL1344 (4/74) (Fig. 5). As the flanking regions of the recombination are nearly identical, the exact locations of recombination boundaries are not known. The position of the single Tn*5* insertion in each chimeric strain is given in Table S2. In Chimera_3 and Chimera_4 the Tn*5* insertions are located at distant positions relative to the Gifsy-1 recombination event, which suggests that more than one transducing particle recombined with the 4/74 receptive bacterium; however, the only shared recombination event in all four chimeric strains is in Gifsy-1_4/74_.

To confirm the 14028s genetic locus that determines strain-specific differences in RtcR activation, we assayed the frequency of cotransduction of KanR with the RtcR activation phenotype (Lac+) into strain LT2, as well as strain 4/74, using as donor strains the 4/74-14028s chimeric strains and selected 14028s single-gene deletion (SGD) mutants (38) whose deletion::KanR positions correlate with Tn*5* positions in the four chimeric strains (Table S2; Fig. 5). P22 lysates were prepared on the donor strains and used to transduce 4/74 or LT2 strains carrying pJK15 (RecA730) and the reporter plasmid, pP*_rsr_-lacZ*; transductants were screened as described for the previous transduction assays. The frequency of Lac+ colonies among all KanR transductants for each donor-recipient pair is listed in Table S2. As expected, Chimera_1 and Chimera_2 were the only chimeras to show linkage of the Tn*5*-encoded KanR gene with the locus conferring the Lac+ phenotype in strains 4/74 and LT2. The associated KanR gene in Chimera_2, which is located immediately downstream of the stop codon of STM14_3218, exhibited 0.95 and 0.96 cotransduction frequencies in 4/74 and LT2, respectively; the associated KanR gene in Chimera_1, which is located approximately 6 kb away from STM14_3218, near the *xis* gene in the prophage recombination region, exhibited 0.27 and 0.08 cotransduction frequencies in 4/74 and LT2, respectively (Fig. 5; Table S2). The SGD mutants in which the KanR cassette is within 8 kb of STM14_3218 in Gifsy-1_14028s_ gave the highest cotransduction frequencies of the RtcR activation phenotype (Lac+), which confirms the cotransduction results with the chimeric strains (Table S2). The lower frequencies of cotransduction observed for some donors with the recipient LT2 reporter strain compared to the recipient 4/74 reporter strain probably reflect reduced recombination between the LT2 genome and donor DNA from the 4/74-14028s chimeric strains or 14028s SGD mutants due to the lower level of sequence identity in the Gifsy-1 prophages (34). These cotransduction assays support that the essential genetic difference between strain 14028s and strains 4/74 and LT2 that permits RecA*-LexA-dependent RtcR activation and expression of the RNA repair operon is the STM14_3218-3220 region of prophage Gifsy-1, which was identified by deletion analysis as required for RtcR activation.

### Production of infectious Gifsy phage particles during the SOS response in *S.* Typhimurium is not affected by the RtcR regulon.

The integral link between induction of the Gifsy-1 prophage and RtcR activation in *S.* Typhimurium strain 14028s suggests that the RtcR regulon (*rsr-yrlBA-rtcBA-dinJ-yafQ* [12]) may play a role in survival of 14028s under stress conditions that lead to lytic growth of Gifsy-1. Although a variety of mechanisms and complex regulatory pathways could be proposed, we address here only one potential outcome if RtcR activation results in a significant portion of a 14028s population under genotoxic stress to abort the induction pathway that leads to lytic replication of Gifsy-1, and possibly Gifsy-2 and Gifsy-3. The titers of Gifsy-1, or all three Gifsy phages, released from cultures of WT, ΔSTM14_3218-3220, and Δ*rtcR* 14028s strains treated with MMC at mid-log-phase growth were determined. The ΔSTM14_3218-3220 mutant, which is defective for activation of RtcR (Fig. 4C), exhibited significant 2-fold and 4-fold higher titers of Gifsy-1 and all three Gifsy phages, respectively, compared to the WT strain; however, the phage titers for the Δ*rtcR* mutant (12) did not differ significantly from those of the WT strain (Fig. S9). Thus, these results support a role for STM14_3218-3220 in Gifsy-1 in moderating the production of viable phage particles upon induction but do not support a role for the RtcR regulon in interrupting induction of the Gifsy-1, -2, or -3 prophages during the SOS response in strain 14028s.

## DISCUSSION

Previous studies have shown that RtcR-dependent expression of the *S.* Typhimurium 14028s RNA repair operon (*rsr-yrlBA-rtcBA*) is stimulated by genotoxic stress that activates RecA and the SOS response; under these conditions a signal ligand, which is predicted to be a cleaved tRNA with a 3′-terminal 2′,3′-cyclic phosphate, is generated that activates RtcR for transcription initiation from the RNA repair operon promoter, *rsrp* (10, 12, 20). In this work, the signal pathway for RtcR activation was dissected, elucidating the genetic determinants for expression of the RNA repair system during the SOS response (summarized in Fig. 1). Utilizing constitutively active variants of RecA and RtcR and a noncleavable variant of LexA in reporter assays for transcription of the RNA repair operon, we demonstrate that nucleic acid-damaging treatments indirectly activate RtcR by facilitating the activation of RecA and that the RecA*-dependent process required for generating the RtcR signal ligand is derepression of LexA-repressed genes (Fig. 2 and 3A; Fig. S2). Supporting this conclusion, a recent study showed that expression of Rsr and accumulation of the potential signal molecule (cleaved tRNAs) upon MMC treatment was significantly reduced in *S.* Typhimurium 14028s mutant strains deleted for *recA* or encoding an uncleavable LexA variant (*lexA3*) (10).

Directed mutagenesis of key genes in each of the LexA-controlled SOS-response DNA-repair pathways revealed that none of the repair pathways are essential for RtcR activation (Fig. 3A). However, another RecA*-LexA-controlled process in *S.* Typhimurium is the induction of stable lambdoid prophages to enter into their infectious cycle. Using strains of 14028s that had been cured of Gifsy-1, Gifsy-2, Gifsy-3, or combinations of these prophages, we determined that any strain lacking Gifsy-1 is unable to activate expression from *rsrp* when the SOS response is induced by RecA730 (Fig. 4A). Surprisingly, despite Gifsy-1 being present in other well-characterized *S.* Typhimurium strains, LT2, SL1344, and 4/74, the RNA repair operon is not activated during the SOS response in these strains (Fig. 4B). Comparisons of the Gifsy-1 genome in various strains of *S.* Typhimurium revealed a specific region of variability within the replication and immunity region (Fig. 5); this region in Gifsy-1_LT2_ and Gifsy-1_SL1344_ bears striking resemblance to Gifsy-3, not Gifsy-1, in 14028s (34). STM14_3218 and STM14_3220, which are within the region of variability, each received two hits in the global Tn*5* insertion mutant screen (Table S1). Directed deletion of STM14_3218 through STM14_3220 confirmed that a feature in this region is essential for RtcR activation (Fig. 4). In addition, the results of sequencing P22 transductants of the 14028s genetic determinant that confers the strain-specific ability to activate RtcR during the SOS response supports that Gifsy-1_14028s_ STM14_3218-3220 is the necessary genomic region when present in *cis* in the prophage (Fig. 5; Table S2). A blastn search with the Gifsy-1_14028s_ nucleotide sequence encoding STM14_3218-3220 on complete genomes in the GenBank nonredundant (NR) database revealed conservation of this region in 86 strains of S. enterica (including 38 strains of serovar Typhimurium, 32 strains of serovar Newport, and two strains of serovars Hissar and Anatum) and one strain of Salmonella bongori. This number of hits represents a small minority of the completed Salmonella genomes in the database. The presence in S. bongori indicates a widespread but infrequent distribution. One gene in this region, STM14_3218, is a member of the same AAA family of ATPases as RtcR. The closest paralog of STM14_3218 in 14028s is the ParA encoded on the pSLT virulence plasmid, although the homology is weak, indicating they are unlikely to have the same role. ParA on the plasmid is a partitioning ATPase, typically utilized by low-copy-number plasmids or bacterial chromosomes to ensure that both daughter cells receive a DNA copy during cell replication (39, 40). ParA usually acts in tandem with ParB, which binds a centromere-like partitioning site on DNA (*parS*). The genes for *parA* and *parB* are typically found in an operon; however, the gene for a ParB homologue could not be identified within the Gifsy-1 genome. Orphan *parA* genes have been previously reported to contribute to the positioning of compartmentalized cellular processes, such as localizing cellulose biosynthesis to the poles in enterobacteria (41). The remaining two genes of the STM14_3218-3220 region are annotated as hypothetical genes, and a putative function could not be ascribed by various analyses (i.e., searches for predicted domains or motifs and structural modeling based on the proposed amino acid sequence). Thus, it remains to be determined how the STM14_3218-3220 region is involved in generating the signal for RtcR activation (Fig. 1).

While the Gifsy-1 STM14_3218-3220 region was identified as the essential prophage-specific determinant, Gifsy-3 also contributes to the RtcR signaling pathway in strain 14028s, as evidenced by the 1.8-fold downregulation of expression from *rsrp* under SOS-inducing conditions in a strain cured of Gifsy-3, compared to the 19- to 116- fold downregulation in strains cured of Gifsy-1 (Fig. 4A; Table 1). The modest level of enhanced expression from *rsrp* in the presence of the Gifsy-3 prophage during the SOS response may be due to increased RecA* activity in response to prophage induction. Our Tn*5* mutagenesis screen identified multiple genes within the Gifsy-3 recombination region whose interruption led to upregulation of the RNA repair operon, including genes involved in prophage excision (*int* and *xis*) and homologous recombination (*recT*, *recE*, and *exoVIII*) (Table S1). Interruption of these genes may lead to greater RecA* activity due to the increased levels of ssDNA as part of escape replication (31) or of recombination intermediates (lambdoid phage replication and recombination functions are reviewed in reference 42).

In addition to processes that are dependent upon RecA*, we identified a bacterial gene that is not directly regulated by RecA but plays an essential role in RtcR-dependent activation of *rsrp*. From our genome-wide Tn*5* mutagenesis screen, we found that *fis* is required for *rsrp* activation within this signaling pathway (Table 1, Fig. 3C) and that RtcR activation exhibits growth phase dependence (Fig. S5) that correlates with the growth phase regulation of *fis* transcripts and Fis protein levels (28, 29). Fis, or factor of inversion stimulation, is a nonspecific nucleoid binding protein that plays a number of roles in the cell, including influencing transcription of a wide subset of genes, mediating site-specific recombination for genomic inversion inherent to flagellar phase variation in Salmonella, contributing to efficient induction of the SOS response, and enhancing excision of phage λ from the E. coli genome (43–46). It is unknown whether Fis contributes to the induction of prophages in Salmonella, but it would be a likely mechanism to influence RtcR activation. Another possibility is that expression from *rsrp* is influenced by the chromosome remodeling activities of Fis, e.g., Fis facilitating an optimal conformation for interactions of enhancer-bound RtcR with Eσ^54^ at the promoter (Fig. 1).

Overall, this study identified essential bacterial and bacteriophage components of a RecA*-LexA-controlled pathway for activation of the RtcR transcriptional regulator of RNA repair genes in *S.* Typhimurium. Although RtcR is widely conserved in the *Proteobacteria*, the pathway for RtcR activation in *S.* Typhimurium strain 14028 requires Gifsy-1 prophage genes that are found in a very limited number of S. enterica serovars and strains. The role for the RtcR regulon in these strains under genotoxic stress conditions, in which prophage are induced to undergo lytic growth, requires further investigation. These studies reveal new avenues for the continued study of RNA repair operon activation and utilization in *S.* Typhimurium and other species.

## MATERIALS AND METHODS

### Growth conditions, strains, plasmids, and bacteriophages.

The wild-type (WT) strain of *S.* Typhimurium was ATCC 14028s; the E. coli WT was MG1655. All other strains utilized in this study were derived from these, unless otherwise indicated. Methods for construction of strains and plasmids are provided in the supplemental materials; see Table S3 for a complete list of strains and plasmids. Bacteriophages P22 HT *int* and P22-H5 (*c2* mutant) were gifts from Timothy Hoover; P22 lysate preparation and transductions were performed as described in reference 47. Lysates of the Gifsy-1, -2, and -3 phages were prepared, and the phage concentration was determined as described in reference 48, with the exception that cultures were treated with 2 μg/mL MMC at mid-log growth and outgrown for 4 h before centrifugation. Unless otherwise stated, bacterial growth was at 37°C in lysogeny broth (LB) (Fisher Scientific, Fair Lawn, NJ), with aeration. Solid medium contained 1.5% agar. Antibiotics and supplements were supplied by Sigma-Aldrich (St. Louis, MO); antibiotics were used for plasmid maintenance and selection of chromosomal markers at the following concentrations: carbenicillin (Car), 100 μg/mL; tetracycline (Tet), 10 μg/mL; kanamycin (Kan), 50 μg/mL; chloramphenicol (Chl), 35 μg/mL.

### Cell growth and induction conditions for expression assays.

For XylE reporter strains transformed with pJK15 (RecA730 expression vector), cultures were grown overnight from a single colony inoculated in LB plus Tet plus 0.2% glucose (to repress the plasmids) and subcultured to an optical density at 600 nm (OD_600_) of 0.05 in 25 mL fresh LB plus Tet (without glucose). For reporter strains that carried other plasmids in addition to pJK15, the appropriate antibiotics were utilized to maintain selection. Cultures were grown to mid-log phase (OD_600_, 0.3 to 0.4), at which point each culture was split into two 10-mL cultures in 50-mL polypropylene conical tubes. For one of each pair, expression of RecA730 was induced with 1 mM IPTG (isopropyl-β-d-thiogalactopyranoside) (Gold Biotechnology, St. Louis, MO); the other remained uninduced as a control. Cultures were returned to the incubator for an additional 90 min; cells were then harvested for XylE assays, which were performed as previously described (12). Results were analyzed using Student’s *t* test analyses (paired *t* test or two-sample *t* test).

To generate RtcR activation conditions in XylE assays with reporter strains that did not contain pJK15 (RecA730), MMC treatment was used. The cultures were prepared as described above with the appropriate antibiotics for any plasmids carried by the reporter strains; one of each of the split cultures at mid-log phase were treated with 3 μM MMC. After 90 min of growth, the MMC-treated and untreated cultures were assayed for XylE activity.

For pCH6 (RtcR_con_), even leaky expression of RtcR_con_ results in extremely high activity at the operon promoter; therefore, 0.2% glucose was always present in the medium prior to induction. Once these cultures reached mid-log phase, they were split equally into two tubes and centrifuged at 3,000 × *g* for 15 min in a swinging bucket centrifuge to pellet the cells. The supernatant was decanted, and cells were resuspended in an equivalent volume of fresh LB plus Tet medium. One of each pair was induced with the addition of 50 μM IPTG; the other remained tightly repressed by adding 0.2% glucose. Cultures were grown for an additional 90 min prior to harvest for the XylE activity assays. XylE assays were performed as previously described (12).

XylE activity assays to assess complementation of RtcR activation in the ΔSTM14_3218-3220 Δ*rsr*::*xylE* reporter strain are described in the Fig. S6 legend.

### Tn*5* mutant library screen for mutations that affect expression from the *rsr* promoter.

A barcoded library of 14028s Tn*5* insertion mutants was generated using the Epicentre EZ-Tn5 <T7/Kan2> insertion kit, and the barcodes mapped to the 14028s genome as previously described (27). Over 40,000 independent insertions were mapped onto the genome. This Tn*5* insertion library was utilized for a genome-wide mutant screen to identify mutations that alter expression from *rsrp*. A single aliquot was subcultured 1:100 in LB plus Kan (60 μg/mL) medium and grown at 37°C with shaking for exactly 8 h. A portion of the outgrowth was added to transducing broth (LB, 1× E salts, 0.2% glucose) with the high-frequency generalized transducing bacteriophage P22 HT *int* at a multiplicity of infection (MOI) of approximately 0.1 PFU/cell. The culture was incubated at 37°C overnight, at which point cellular debris was removed by centrifugation and the supernatant was sterilized with the addition of chloroform to generate a P22 transducing lysate consisting of the entire Tn*5* library.

WT 14028s cells were cotransformed with pJK15 (RecA730) and pJK19 (*rsrp*-*lacZY*). Overnight cultures were mixed in equal volume with a 10^−2^ dilution of the Tn*5* library P22 lysate and incubated at 37°C for 30 min to allow attachment of the P22 phage, and then 10^0^ and 10^−1^ dilutions were plated on inducing indicator medium (LB agar containing 10 mM EGTA, 80 μg/mL X-Gal, 0.5 mM IPTG, 0.02% glucose, 10 μg/mL Tet, 35 μg/mL Chl, and 50 μg/mL Kan). The uninfected culture and the phage lysate were plated on the inducing indication medium as negative controls. Several independent transductions were performed; for each transduction, all of the phage-cell mix and the 10^−1^ dilution were plated in 50-μL aliquots. Plates were incubated at 30°C for 48 h and were screened for the appearance of white or dark blue colonies. Colonies of interest were streaked onto EBU plates (25 g/L LB, 0.5% K_2_HPO_4_, 0.8% glucose, 0.00125% Evans blue, 0.0025% sodium fluorescein) to remove contaminating phage and were checked for sensitivity to infection by P22-H5 (*c2* mutant) to confirm that transductants were not P22 lysogens (47). To reduce ambiguity of the results, phage-free colonies from the EBU plates were screened a second time by patching onto inducing indicator medium without EGTA. Patches that grew white or darker blue than the WT control were subsequently inoculated in LB broth and grown overnight; mutants were pooled and processed as follows for sequencing to identify the locations of the mutations.

**(i) Library preparation and sequencing.** Genomic DNA was prepared from pooled mutant samples using the cetyltrimethylammonium bromide (CTAB)-based extraction method (49). DNA pellets were resuspended in 100 μL Tris buffer (10 mM Tris, pH 8.5). Samples were quantified using a Qubit 3.0 fluorometer, following the standard protocol for the double-stranded DNA (dsDNA) broad-range (BR) assay. Genomic DNA (gDNA) quality was assessed by digest with EcoRI-HF followed by gel electrophoresis (0.8% agarose) of the cut and uncut samples. The genomic DNA from the pooled mutants was then used in a nested PCR regimen, previously described in detail in reference 27. Oligonucleotides 65 and 66 were used to amplify the right flanking regions containing an 18-base barcode; a second PCR with oligonucleotides 67 and 68 amplified the (already enriched) right flank of the transposon insertion site (Table S4). Illumina sequencing proceeded with oligos 69 and 70 (Table S4) for a single indexed run with a read length of 25 bases. The first 18 bases of the raw sequencing data represent the unique 18-mer tag for each Tn*5* mutant; these were analyzed as described (27) and mapped to their previously determined position within the 14028s genome.

**(ii) Confirming the role for genes identified in the Tn*5* screen in *rsr* promoter activation.** Single-gene deletion mutants corresponding to the Tn*5* insertion mutants were obtained from the BEI 14028s single-gene deletion mutant library (38); see Table S3.

Strains to be used in beta-galactosidase assays (Table S3; strains JEK72 through JEK82) were generated by transferring deletions into a clean 14028s genetic background by P22 transduction, as described for the SOS-response reporter strains in the supplemental methods; these strains additionally carried pJK15 (*lacp-recA730*) and pJK19 (*rsrp*-*lacZY*) for activation of *rsrp* and expression of LacZ, respectively. Mutations were confirmed by PCR with gene-flanking primers (Table S4; oligonucleotides 71 through 88).

Several mutant strains were further examined by generating chromosomal Δ*rsr*::*xylE* reporter strains for XylE assays. Deletions were moved into strain JEK17 by P22 transduction (Table S3; strains JEK101 through JEK128) and were checked by PCR with the same gene-flanking primers as described above.

**(iii) Beta-galactosidase assays.** Strains carrying pJK15 (*lacp-recA730*) and pJK19 (*rsrp*-*lacZY*) were grown, induced, and treated following the same protocol as described above. Beta-galactosidase assays were conducted using established protocols (50). Results were analyzed using Student’s *t* test analyses (paired *t* test or two-sample *t* test).

### P22 transduction assays to identify genomic regions of 14028s that allow activation of RtcR.

*S.* Typhimurium strain 4/74, which does not activate RtcR upon induction of the SOS response, was transformed with pP*_rsr_-lacZ* and pJK15 (RecA730) to create a reporter strain for RtcR activation. This reporter strain was transduced with a P22 HT *int* lysate prepared on the Tn*5* insertion library in 14028s and plated on inducing indicator medium as described above for the Tn*5* insertion mutant screen. Blue KanR transductants were streaked on EBU plates and checked for sensitivity to P22-H5 as described above. These 4/74 transductants were confirmed for RecA730-dependent *lacZ* expression from the reporter plasmid pP*_rsr_-lacZ* by patching on both inducing indicator medium and noninducing indicator medium that contained no IPTG and 0.2% glucose; four isolates with the desired phenotype (no. 102, 860, 957, and 1458, which are designated Chimera_1, _2, _3, and _4, respectively, here) were chosen to prepare genomic DNA for sequencing. The library was created using the Perkin Elmer NEXTFLEX rapid DNA kit v2 following the manufacturer’s instructions and sequenced to a depth of over 100× using paired-end sequencing, PE100. The genomes were assembled and compared to the GenBank sequences of the 14028 and SL1344 genomes using CLCBio (Qiagen).

**(i) P22 cotransduction assays.** The 4/74 reporter strain was transduced with P22 HT *int* lysates prepared on the indicated SGD mutants using the protocols described above. The frequency of cotransduction of the *kanR* cassette of each SGD with the genomic region required for RtcR activation (blue colony screen) was assessed with at least 3 independent transduction assays for each SGD.
