# Supplementary material for: Strain-Specific Gifsy-1 Prophage Genes Are Determinants for Expression of the RNA Repair Operon during the SOS Response in Salmonella enterica Serovar Typhimurium
Source: J Bacteriol. 2023 Jan 9;205(1):e00262-22. doi: 10.1128/jb.00262-22 (PMC9879122; doi:10.1128/jb.00262-22)
Supplement: Supplemental file 1 — Supplemental methods, Tables S1 to S4, and Fig. S1 to S9. Download jb.00262-22-s0001.pdf, PDF file, 1.1 MB [file jb.00262-22-s0001.pdf]

SUPPLEMENTAL MATERIALS: METHODS, TABLES AND FIGURES.

Supplemental Methods- Strain and Plasmid Construction

All strains and plasmids are listed in **Table S3;** the name and sequence for all the numbered oligonucleotides are given in **Table S4.**

Strains for assaying role of SOS response pathways. Construction of the reporter strains JEK17 (Δ*rsr*::*xylE*), JEK26 (JEK17 Δ*recA*::*kan*), and JEK41 (JEK17 Δ*rtcR*::*kan*) was described previously (1). To generate the reporter strain JEK19 (JEK17 Δ*tisB*::*kan*) a PCR fragment consisting of a *kan* cassette flanked by sequence that is homologous to the regions that flank *tisB* on the chromosome was amplified from the template pKD4 (oligos 1 & 2) using OneTaq polymerase (New England Biolabs, Ipswich, MA). λ-Red recombination (2) was used to recombine the fragment into the 14028s chromosome, replacing *tisB* (start to stop codon) with *kan*. The high-frequency generalized transducing bacteriophage P22 HT 105/1 *int-201* (P22 HT *int*) was used to transfer the Δ*tisB*::*kan* deletion into strain JEK17, and the deletion was confirmed by PCR (oligos 3 & 4). The Δ*rsr*::*xylE* reporter strains JEK34 (Δ*recR*::*kan*), JEK36 (Δ*uvrB*::*kan*), JEK38 (Δ*ruvC*::*kan*), JEK40 (Δ*ruvA*::*kan*), JEK44 (Δ*umuC*::*kan*), JEK46 (Δ*umuD*::*kan*), JEK48 (Δ*recO*::*kan*), JEK50 (Δ*sulA*::*kan*), JEK89 (Δ*polB*::*kan*), JEK90 (Δ*dinP*::*kan*), JEK93 (Δ*hslV*::*kan*), JEK115 (Δ*recB*::*cam*), and JEK116 (Δ*recC*::*kan*) were all generated using standard P22 transduction with the bacteriophage P22 HT *int* (3); the donor mutant strains were obtained from the BEI 14028s single gene deletion (SGD) library (4) and the recipient strain was JEK17. JEK49 (14028s Δ*sulA*::*kan*) was generated similarly using WT 14028s as the recipient strain; JEK56 (14028s Δ*sulA*) was constructed by transforming JEK49 with pCP20 (2) to remove the *kan* cassette. Transductants were isolated from contaminating phage on EBU plates (25 g/L LB, 0.5% K_2HPO_4, 0.8% glucose, 0.00125% Evan's Blue, 0.0025% sodium fluorescein) and were checked for sensitivity to infection by P22-H5 (*c2* mutant), confirming transductants were not P22 lysogens (3). All strains were confirmed for the appropriate deletions by PCR analysis using gene-flanking primers (*recR* (oligos 5&6), *uvrB* (7&8), *ruvC* (9&10), *ruvA* (11&12), *umuC* (13&14), *umuD* (15&16), *recO* (17&18), *sulA* (19&20), *polB* (21&22), *dinP* (23&24), *hslV* (25&26), *recB* (27&28), *recC* (29&30)). Strain JEK112 (*lexA33::cam*) was generated similarly using P22 transduction with lysate grown on strain TT23994 (LT2 *lexA33*::*cam* (lexA3IND-)) (5), and the substitution was confirmed by PCR with gene-flanking primers (oligos 31 & 32).

Prophage-cured and prophage-gene deletions reporter strains. XylE reporter strains that were cured of different combinations of prophages were generated using P22 transduction to transfer the *rsrp-xylE-kan* reporter fusion from JEK12 (14028s Δ*rsr*::*xylE-kan*) into MA5958 (14028s parent strain), MA5973 (Gifsy-1[-]), MA6275 (Gifsy-2[-]), MA6051 (Gifsy-3[-]), MA5975 (Gifsy-1[-] Gifsy-2[-]), and MA6052 (Gifsy-1[-] Gifsy-2[-] Gifsy-3[-]) (6, 7); the *kan* cassette was removed by transforming strains with pCP20 (2), generating strains JEK87, JEK83, JEK84, JEK85, JEK86, and JEK60, respectively. Replacement of *rsr* with the reporter gene and excision of *kan* were verified by PCR with *rsr*-flanking primers (oligos 33 & 34), absence of prophages was confirmed using primers that flank each prophage genome [Gifsy-1 (oligos 35 & 36), Gifsy-2 (oligos 39 & 40), Gifsy-3 (oligos 43 & 44)], and presence of prophages was confirmed using primers that anneal to unique sites within each prophage [Gifsy-1 (oligos 37 & 38), Gifsy-2 (oligos 41 & 42), Gifsy-3 (oligos 45 & 46)].

Reporter variants of 14028s that were deleted for Gifsy-1 phage genes were constructed using λ-Red recombination (2). STM14_3177 and STM14_3211 were deleted from start codon to stop codon; for ΔSTM14_3218-3220, the putative operon was deleted from the start codon of STM14_3218 to the stop codon of STM14_3220. PCR was used to amplify the *kan* cassette from pKD4, with the addition of flanking sequences homologous to the regions that flank STM14_3177 (oligos 93 & 94), STM14_3211 (oligos 89

& 90) or STM14_3218-3220 (oligos 91 & 92) on the chromosome. The resulting mutants (JEK133, JEK131, and JEK132, respectively) were used to propagate P22 phage, and the mutations were transferred into JEK17 using P22 transduction, as described. The *kan* cassette was excised after transformation with, and subsequent loss of, pCP20 to generate strains JEK136, JEK134, and JEK135, respectively. Mutant strains were confirmed by PCR using gene/operon-flanking primers (oligos 95-98).

E. coli *and other* Salmonella *reporter strains.* The *E. coli* reporter strain ACW1 was generated by λ-Red recombination. The *xylE-kan* reporter fusion was amplified from JEK12 genomic DNA, with the addition of flanking regions of homology to the regions that flank *rtcB* on the MG1655 chromosome (oligos 47 & 48), and this fragment was used to transform MG1655 expressing the λ-Red genes from pKD46 (2). The *kan* cassette was subsequently removed by transforming cells with pCP20 (2), producing strain ACW2 (MG1655 Δ*rtcB*::*xylE*). Proper insertion of the reporter fusion and removal of *kan* were confirmed by PCR with gene-flanking primers (oligos 49 & 50).

The Δ*rsr*::*xylE* chromosomal reporter fusion was constructed in *S*. Typhimurium strains LT2, SL1344, and 4/74 to generate reporter strains JEK61 (LT2), JEK64 (SL1344), and JEK114 (4/74). The Δ*rsr*::*xylE-kan* deletion-insertion mutation was transferred by P22 transduction with lysate grown on strain JEK12, and the *kan* cassette was subsequently removed upon transformation of the strains with pCP20 (8). Insertion of the reporter and loss of the *kan* cassette were confirmed by PCR with *rsr* flanking primers (oligos 33 & 34).

Plasmid construction. PCR amplification for plasmid construction used OneTaq DNA polymerase or Q5 High-Fidelity DNA polymerase, and ligation reactions used T4 DNA ligase, according the manufacturers recommended protocols (NEB, Ipswich, MA). Restriction enzymes were purchased from NEB. Plasmid products were transformed into chemically competent DH5α; for use in *Salmonella*, plasmids were replicated in the restriction$^-$ modification$^+$ LT2 strain MS1868 prior to transformation into relevant strains.

Plasmid pJK14 was constructed by PCR amplifying the 14028s *recA* gene, with the addition of flanking NdeI and XhoI cut sites (oligos 51 & 54). The amplicon was digested with NdeI and XhoI, and the NdeI-XhoI fragment was introduced into the expression vector pSRK-Tc (9). pJK15 was constructed similarly, except that overlap extension PCR (10) was first used to introduce the single base pair mutation that converts the Glu^{39} GAA codon to a Lys AAA codon. The *recA* gene was amplified from 14028s gDNA in two pieces using oligos 51 & 52 and 53 & 54**)**; oligos 52 and 53 are direct complements that introduce the single G → A mismatch. The two fragments were then used in a subsequent PCR reaction, primed by their overlapping ends to generate the complete *recA730* allele flanked by NdeI and XhoI for introduction into pSRK-Tc (11).

For pCH6, a PCR product encoding a truncated copy of *S.* Typhimurium RtcR beginning at Leu^{179} was generated with the addition of flanking NdeI and XhoI cut sites (oligos 55 & 56) and TOPO cloned into pCR2.1 to make pCH4; the NdeI-XhoI fragment from pCH4 was then introduced into pSRK-Tc. Plasmid constructs were confirmed by Sanger sequencing (oligos 57 & 58) (Genewiz, South Plainfield, NJ).

To construct pJK19, a single-copy reporter plasmid in which *rsrp* is fused to *lacZY*, the regulatory region encompassing the transcription start site (TSS) of *rsr* through 634 bp of the N-terminal domain-encoding region of *rtcR* was PCR amplified from 14028s genomic DNA with the addition of NotI and HindIII cut sites (oligos 59 & 60). The NotI-HindIII con promoter region of pAG620 (12) was replaced with the NotI-HindIII *rsrp* fragment; ligations were additionally digested with KpnI, which cuts within the

con promoter region to prevent re-ligation of pAG602. Appropriate expression of LacZ was confirmed by plating *S.* Typhimurium WT, Δ*rtcR*, and Δ*rpoN* strains expressing RecA730 on X-Gal-containing medium.

pJK21 was generated by PCR amplifying the STM14_3218-3220 putative operon sequence from 14028s genomic DNA, with the addition of flanking NheI and HindIII cut sites (oligos 61 & 62). Amplicons were digested with NheI and HindIII, and the fragment was introduced into pBAD30 (13). The resulting plasmid was confirmed by Sanger sequencing at Genewiz (South Plainfield, NJ) using standard pBAD sequencing primers and full-length plasmid sequencing at Plasmidsaurus (Eugene, OR). Plasmids pAK101, pAK102 and pAK103 were constructed by PCR amplifying 14028s STM14_3218, STM14_3218-3220, and STM14_3219-3220 with the addition of flanking NdeI and XhoI cut sites (oligos 99 & 100, 99 & 101, 102 & 101, respectively). The amplicons were digested with NdeI and XhoI, and the NdeI-XhoI fragments were introduced into pSRK-Tc (9). The resulting plasmids were confirmed by full-length plasmid sequencing at Plasmidsaurus (Eugene, OR).

For the construction of pP_{rsr}-*lacZ*, a fragment containing 373 bp of the *rtcR*-*rsr* intergenic sequence and part of *rtcR* was generated by PCR amplification from *S.* Typhimurium LT2 genomic DNA with the addition of flanking XbaI and BamHI restriction sites (oligos 63 &64). The amplicon was inserted into pGEM-T (Promega); the resulting plasmid was digested with XbaI and BamHI and the XbaI-BamHI fragment containing the *rsr* promoter was isolated by agarose gel extraction and introduced into the MCS of p16R (pACYC184 with the BamHI to SalI region replaced by the *E. coli lacZ* gene; provided by Timothy Hoover).

Tables-

Table S1. Bacterial and prophage genes identified in Tn5 mutagenesis screen.

Gene Position	Tn5 Insertions Positions	Locus (Gene)	Function	Tn5 Screen Result
214578-215996	215464, 215724, 215992	STM14_0218 (*pcnB*)	Poly-A polymerase	White
508798-509070	508726[a]	STM14_0534 (*hupB*)	Transcriptional regulator HU subunit beta; histone-like DNA binding protein	Blue+
1284622-1285764	1284994, 1285129, 1285234	STM14_1417	Gifsy-3 integrase (*int*)	Blue+
1285754-1285990	1285933	STM14_1418	Gifsy-3 excisionase (*xis*)	Blue+
1286040-1286591	1286150	STM14_1419	Gifsy-3 Eaa protein	Blue+
1286588-1286920	1286818	STM14_1420	Gifsy-3 hypothetical protein	Blue+
1286913-1287239	1287143	STM14_1421	Gifsy-3 hypothetical protein	White
1287269-1288099	1287393, 1287507, 1287846	STM14_1422	Gifsy-3 RecT homolog	Blue+
1288092-1290446	1289115, 1289439, 1290345	STM14_1423	Gifsy-3 exodeoxyribonuclease VIII	Blue+
1290505-1290804	1290642	STM14_1424	Gifsy-3 RecE homolog	Blue+
2415829-2416881	2416140	STM14_2796 (*apbE*)	Thiamine biosynthesis lipoprotein	White
2416996-2418132	2418007	STM14_2797 (*ompC*)	OmpC outer membrane porin	White
2418383-2418563	2418591	STM14_2799 (*micF*)	Regulatory RNA; represses expression of RpoS	White

2418861-2421530	2419466, 2419658, 2421192	STM14_2800 (*yojN*/*rcsD*)	Phosphotransfer intermediate in RcsBC two-component regulatory system	White
2821795-2822634	2821843, 2822337	STM14_3218	Gifsy-1 AAA ATPase family	White
2823013-2823675	2823035, 2823036	STM14_3220	Gifsy-1 hypothetical protein	White
3085732-3086723	3086719	STM14_3526 (*rpoS*)	RpoS sigma factor	Blue+
3086786-3087919	3086953	STM14_3527 (*nlpD1*)	Outer membrane lipoprotein	Blue+
3570161-3570457	3570338	STM14_4083 (*fis*)	Nucleoid-binding protein, regulates transcription of *rpoS;* enhances excision of bacteriophage λ	White
3698566-3700149	3698602, 3698719, 3698747, 3698894, 3699171, 3699295, 3699611	STM14_4241 (*rtcR*)	Regulator of RNA repair operon	White
4400290-4400562	4400391	STM14_5011 (*hupA*)	Transcriptional regulator HU subunit alpha; histone-like DNA binding protein	Blue+
4617786-4619066	4618231	STM14_5243 (*hflX*)	Putative GTPase; part of high frequency of lysogenization locus	White
4619281-4620540	4619587	STM14_5244 (*hflK*)	FtsK protease regulator; part of high frequency of lysogenization locus	White

[a] Tn5 insertion occurred in the intergenic/regulatory region directly upstream of *hupB*.

Table S2. Co-transduction of KanR markers with the RtcR-activation Locus (Lac+).

Donor strain	Recipient *S*. Typhimurium Reporter Strain		Position of Kan^R in 14028s Chromosome
	**4/74 (pP$_{rsr}$-*lacZ*)**	**LT2 (pP$_{rsr}$-*lacZ*)**	
SGD_1153*	0.00 ± 0.00	0.00 ± 0.00	1,066,349
Chimera_4	0.00 ± 0.00	0.00 ± 0.00	2,056,734
SGD_2398	0.00 ± 0.00	0.00 ± 0.00	2,075,941
SGD_3169	0.00 ± 0.00	**0.04 ± 0.04**	2,785,434
SGD_3204	**0.73 ± 0.08**	**0.56 ± 0.07**	2,815,110
Chimera_2	**0.95 ± 0.08**	**0.96 ± 0.05**	2,822,632
Chimera_1	**0.27 ± 0.14**	**0.08 ± 0.04**	2,828,849
SGD_3226	**0.39 ± 0.15**	**0.06 ± 0.03**	2,828,879
SGD_3227	**0.52 ± 0.03**	**0.18 ± 0.05**	2,829,159
SGD_3242	**0.03 ± 0.03**	**0.06 ± 0.03**	2,839,091
Chimera_3	0.00 ± 0.00	0.00 ± 0.00	3,281,213
No P22 lysate	0.00 ± 0.00	0.00 ± 0.00	-

*control: KanR cassette replaces hypothetical gene within the regulation region of prophage Gifsy-2

Table S3. Strains and plasmids used in this study.

**Strain**	**Genotype**	**Source**
***Salmonella* Typhimurium Strains**		
14028s	Wild-type	ATCC
ATCC 700720	LT2	ATCC
MS1868	LT2 *leuA*414 *hslD* Fels2	(14)
MZ1601	4/74	(15)
SB300A#1	SL1344 *rpsL hisG aadA*::*araC*-PBAD-T7 pol	(16)
TT23994	LT2 *recN557*::MudJ	(5); courtesy of John Roth
DJS101	14028S Δ*rtcR*	(1)
JEK12	14028s Δ*rsr*::*xylE-kan*	(1)
JEK17	14028s Δ*rsr*::*xylE*	(1)
JEK26	JEK17 Δ*recA*::*kan*	(1)
JEK41	JEK17 Δ*rtcR*::*kan*	(1)
JEK61	ATCC 700720 (LT2) Δ*rsr*::*xylE*	This study
JEK64	SB300#1 (SL1344) Δ*rsr*::*xylE*	This study
JEK114	MZ1601 (4/74) Δ*rsr*::*xylE*	This study
**14028s SOS Response Mutants**		
SGD_recR	14028s Δ*recR*::*kan*	(4); BEI Resources, ATCC
SGD_uvrB	14028s Δ*uvrB*::*kan*	(4); BEI Resources, ATCC
SGD_ruvC	14028s Δ*ruvC*::*kan*	(4); BEI Resources, ATCC
SGD_ruvA	14028s Δ*ruvA*::*kan*	(4); BEI Resources, ATCC
SGD_umuC	14028s Δ*umuC*::*kan*	(4); BEI Resources, ATCC
SGD_umuD	14028s Δ*umuD*::*kan*	(4); BEI Resources, ATCC
SGD_recO	14028s Δ*recO*::*kan*	(4); BEI Resources, ATCC

SGD_sulA	14028s Δ*sulA*::*kan*	(4); BEI Resources, ATCC
SGD_polB	14028s Δ*polB*::*kan*	(4); BEI Resources, ATCC
SGD_dinP	14028s Δ*dinP*::*kan*	(4); BEI Resources, ATCC
SGD_hslV	14028s Δ*hslV*::*kan*	(4); BEI Resources, ATCC
SGD_recB	14028s Δ*recB*::*cam*	(4); BEI Resources, ATCC
SGD_recC	14028s Δ*recC*::*kan*	(4); BEI Resources, ATCC
TT22964	LT2 *lexA33*::*cam* (lexA3IND-)(sw)	(5); courtesy of John Roth
JEK19	JEK17 Δ*tisB*::*kan*	This study
JEK34	JEK17 Δ*recR*::*kan*	This study
JEK36	JEK17 Δ*uvrB*::*kan*	This study
JEK38	JEK17 Δ*ruvC*::*kan*	This study
JEK40	JEK17 Δ*ruvA*::*kan*	This study
JEK44	JEK17 Δ*umuC*::*kan*	This study
JEK46	JEK17 Δ*umuD*::*kan*	This study
JEK48	JEK17 Δ*recO*::*kan*	This study
JEK49	14028s Δ*sulA*::*kan*	This study
JEK50	JEK17 Δ*sulA*::*kan*	This study
JEK56	14028s Δ*sulA*	This study
JEK89	JEK17 Δ*polB*::*kan*	This study
JEK90	JEK17 Δ*dinP*::*kan*	This study
JEK93	JEK17 Δ*hslV*::*kan*	This study
JEK112	JEK17 *lexA33::cam*	This study
JEK115	JEK17 Δ*recB*::*cam*	This study
JEK116	JEK17 Δ*recC*::*kan*	This study
**14028s Prophage-Cured Strains and Prophage-Gene Deletion Strains**		
MA5958	ATCC14028s (parent strain for phage-cured strains from Bossi laboratory)	(7); courtesy of Lionello Bossi
MA5973	14028s Gifsy-1[-]	(6); courtesy of L. Bossi
MA5975	14028s Gifsy-1[-] Gifsy-2[-]	(6); courtesy of L. Bossi

MA6051	14028s Gifsy-3[-]	(7); courtesy of L. Bossi
MA6052	14028s Gifsy-1[-] Gifsy-2[-] Gifsy-3[-]	(7); courtesy of L. Bossi
MA6275	14028s Gifsy-2[-]	(6); courtesy of L. Bossi
JEK60	MA6052 Δ*rsr*::*xylE*	This study
JEK83	MA5973 Δ*rsr*::*xylE*	This study
JEK84	MA6275 Δ*rsr*::*xylE*	This study
JEK85	MA6051 Δ*rsr*::*xylE*	This study
JEK86	MA5975 Δ*rsr*::*xylE*	This study
JEK87	MA5958 Δ*rsr*::*xylE*	This study
JEK134	JEK17 ΔSTM14_3211	This study
JEK135	JEK17 ΔSTM14_3218-3220	This study
JEK136	JEK17 ΔSTM14_3177	This study
**Tn5 Mutagenesis Screen and 14028s Deletion Mutants**		
14028s 40k Tn5 library	40k complexity library of barcoded 14028s Tn5 insertion mutants	(17)
SGD_pcnB	14028s Δ*pcnB*::*kan*	(4); BEI Resources, ATCC
SGD_hupA	14028s Δ*hupA*::*kan*	(4); BEI Resources, ATCC
SGD_fis	14028s Δ*fis*::*kan*	(4); BEI Resources, ATCC
SGD_hflX	14028s Δ*hflX*::*kan*	(4); BEI Resources, ATCC
SGD_rcsD	14028s Δ*rcsD*::*kan*	(4); BEI Resources, ATCC
SGD_hupB	14028s Δ*hupB*::*kan*	(4); BEI Resources, ATCC
SGD_rpoS	14028s Δ*rpoS*::*kan*	(4); BEI Resources, ATCC
SGD_ppk	14028s Δ*ppk::kan*	(4); BEI Resources, ATCC
SGD_ompC	14028s Δ*ompC::kan*	(4); BEI Resources, ATCC
JEK72	14028s Δ*hflX*::*kan* (isogenic WT background)	This study
JEK75	14028s Δ*hupB*::*kan* (isogenic WT background)	This study
JEK77	14028s Δ*rcsD*::*kan* (isogenic WT background)	This study
JEK79	14028s Δ*fis*::*kan* (isogenic WT background)	This study
JEK81	14028s Δ*pcnB*::*kan* (isogenic WT background)	This study

JEK82	14028s Δ*hupA*::*kan* (isogenic WT background)	This study
JEK101	JEK17 Δ*fis*	This study
JEK102	JEK17 Δ*pcnB*	This study
JEK104	JEK17 Δ*rpoS*	This study
JEK105	JEK17 Δ*ppk*	This study
JEK122	JEK17 Δ*ompC*	This study
JEK126	JEK17 Δ*hflX*	This study
JEK127	JEK17 Δ*hupA*	This study
JEK128	JEK17 Δ*rcsD*	This study
**14208s Single Gene Deletion Strains for Transduction Mapping**		
SGD_1153	14028s ΔSTM14_1153::*kan*	(4); BEI Resources, ATCC
SGD_2398	14028s ΔSTM14_2398::*kan*	(4); BEI Resources, ATCC
SGD_3169	14028s ΔSTM14_3169::*kan*	(4); BEI Resources, ATCC
SGD_3204	14028s ΔSTM14_3204::*kan*	(4); BEI Resources, ATCC
SGD_3226	14028s ΔSTM14_3226::*kan*	(4); BEI Resources, ATCC
SGD_3227	14028s ΔSTM14_3227::*kan*	(4); BEI Resources, ATCC
SGD_3242	14028s ΔSTM14_3242::*kan*	(4); BEI Resources, ATCC
***E. coli* Strains**		
DH5α		New England Biolabs
MG1655		Courtesy of Sidney Kushner
ACW1	MG1655 Δ*rtcB*::*xylE-kan*	This study
ACW2	MG1655 Δ*rtcB*::*xylE*	This study
**Plasmids**		
p16R	pACYC184 with *lacZ* replacing BamHI-SalI region, Chl^R	Courtesy of Timothy Hoover
pAG620	Single-copy-number plasmid with constitutive *tac* promoter (con) upstream of *lacZY*, Chl^R	(12)
pAK101	pSRK-Tc with NdeI-XhoI STM14_3218 fragment, amplified from 14028s genomic DNA	This study

pAK102	pSRK-Tc with NdeI-XhoI STM14_3218-3220 fragment, amplified from 14028s genomic DNA	This study
pAK103	pSRK-Tc with NdeI-XhoI STM14_3219-3220 fragment, amplified from 14028s genomic DNA	This study
pBAD30	Derivative of pACYC-184, expression vector with arabinose-inducible p*araBAD* promoter, Car^R	(13); courtesy of Jorge Escalante
pCH4	pCR2.1 with truncated *rtcR* fragment, from Leu179 codon to stop codon, to produce $RtcR_{con}$	This study
pCH6	pSRK-Tc with NdeI-XhoI fragment from pCH4	This study
pCP20	Temperature-sensitive origin, thermal induced FLP recombinase expression vector, Car^R	(8)
pCR2.1	TA cloning vector, Car^R Kan^R	Invitrogen; Carlsbad, CA
pJK14	pSRK-Tc with NdeI-XhoI *recA* fragment, amplified from 14028s genomic DNA	This study
pJK15	pSRK-Tc with NdeI-XhoI *recA730* fragment, amplified from 14028s genomic DNA and modified by overlap extension PCR	This study
pJK19	pAG620 with con promoter region swapped with NotI-HindIII fragment containing *rsr*p and upstream regulatory elements	This study
pJK21	pBAD30 with EcoRI-HindIII STM14_3218-3220 fragment, amplified from 14028s genomic DNA	This study
pKD4	*kan* template with FLP recognition target sites for λ-Red recombination, Kan^R	(2)
pKD46	Expresses λ-Red genes *γ*, *β*, and *exo* from an arabinose-inducible p*araB* promoter, Car^R	(2)
pP_{rsr}-*lacZ*	p16R with 373 bp fragment containing *rtcR-rsr* intergenic sequence in XbaI-BamHI of MCS	This study
pSRK-Tc	Derivative of pBBR1, expression vector containing a reengineered *lacI*^{q}-*lac* promoter-operator complex for tight repression in absence of inducer, Tet^R	(9); courtesy of Stephen Farrand

Table S4. Oligos used in this study.

#	Oligo Name	Sequence (5' → 3')
**SOS Response Reporter Strains Construction**		
1	tisB-Red-F	ACGTCGGCTGGCAGTGCTCCTTAACCACAGGAGACGCGTTG TGTAGGCTGGAGCTGCTTC
2	tisB-Red-R	GCGGTTGCCCGCTCCCCTTCGGTGCGGCTTGAATCTGAACAT ATGAATATCCTCCTT
3	cktisB-F	GTCAACACGCACACTATTCCT
4	cktisB-R	AAAGACCAGCGGATAAGGTGA
5	ckrecR-F	ACCCAAGCCTGCTGGAAGATG
6	ckrecR-R	CGCATCTGAGGCGTTAGAGATGAG
7	ckuvrB-F	AAACTTTCATCATCCGACTTGAC
8	ckuvrB-R	TTTCATAGCCGAGGAGAGAGG
9	ckruvC-F	CGGTGAGATCTCTGATGAGGTG
10	ckruvC-R	CCTGTGGCATCATCCCAATTTAG
11	ckruvA-F	GAAGGGAAGGCCATTGAGATAG
12	ckruvA-R	GCCGACATATTCCGCCAATAAC
13	ckumuC-F	TCGTTTCTCATCCCAGCTCAAC
14	ckumuC-R	CGCACAACCATTAACGCATGG
15	ckumuD-F	GAGAAACTTACAAACGAAGCGACG
16	ckumuD-R	GGTTCGACATATCAGCGTAAAGC
17	ckrecO-F	TGGCGTCTGAAATCATCCGTG
18	ckrecO-R	TTTGCGTTTCATCGGCATCG
19	cksulA-F	CCATTAGCCACCATCGCAAAG
20	cksulA-R	CAAGTGTGAACTCCGTCAGAC
21	ckpolB-F	CGAAACCCGCTTCTTACTGC
22	ckpolB-R	CCCGTGGACGGGAAAAGTAA
23	ckdinP-F2	ACGGCAGTTTTTCGCAAGTC

24	ckdinP-R2	CTGCGCCAATAGGATAGCGA
25	ckhslV-F2	AACCAGAAAGTCACCGGCAATG
26	ckhslV-R2	TCCGGGTTGACCAGTTTAGC
27	ckrecB-F	GCATCCCGTTTAAGCAAGGATTTCG
28	ckrecB-R	CACCACATGCGATTCAGGTAGAG
29	ckrecC-F	GTTTAGCCTCCCGGAAGTTTTAGTG
30	ckrecC-R	CGAAGAGCGCATCATAGTCGAAG
31	cklexA-F	CCTGGGGCATCCTGTTATGG
32	cklexA-R	CGCCTTATCCGGCCTACAAA
33	ckSTM3521-F	GCAGATCGATACACACCAGGC
34	ckSTM3521-R	TTTAATGACTACCGTGCAGGC
**Phage-Cured Reporter Strain Construction**		
35	Gifsy1-FlankF	AATCCAGCGTCACACTCTGGG
36	Gifsy1-FlankR	ACCACGCATACCATTGTTGTGC
37	Gifsy1-IntF	GTGCTGTATCCAGTAGAAGCC
38	Gifsy1-IntR	TCAGTCAGACACTACCATGCG
39	Gifsy2-FlankF	ATGAACCAGCTCACTGATCACG
40	Gifsy2-FlankR	TTTCGGCATCGAGGTCAAAGG
41	Gifsy2-IntF	TCGTCTTGATGACGCCAGTCA
42	Gifsy2-IntR	TCCATTGTTCTCCTCCAGAGTC
43	Gifsy3-FlankF	ACGAATGCGCGTTGTTTGAAGC
44	Gifsy3-FlankR	AGGTGAATCTGCAAGCTTCTCC
45	Gifsy3-IntF	CTTTGCCTTCGATGGTGATACT
46	Gifsy3-IntR	CCGCCGTCAGTTATTCGTTAT
***E. coli* Reporter Strain Construction**		
47	rtcB-xylE	TGCAATTATCAGGACAGCAAACAACGAAAAGAGAAAAACA AAATGAACAAAGGTGTAATGCGACC
48	rtcB-kanR	TTCGCCCTGTGCGCCATCCAGCGCAATCATCCTTTTCATCAT CATATGAATATCCT CTTA

49	Ec-ckrtcB-F	TATCAGGACAGCAAACAACGA
50	Ec-ckrtcB-R	CGACTTCAAGCGAGACGGTAT
**Plasmid Construction**		
51	RecA-NdeI-F	ATCACTCATATGGCTATCGACGAAAACAAACAGAAAGCG
52	RecA-E39K-R	AACCGGTGGAGATAGTTTTCACATCCATAGAACGGTC
53	RecA-E39K-F	CGTTCTATGGATGTGAAAACTATCTCCACCGGTTCG
54	RecA-XhoI-R	ATCACTCTCGAGTTAAAAATCTTCGTTGGTTTCTGCAACG
55	RtcRcon-NdeI	ACGCATATGCTCAACTTCCTGAAGTCCGGCATTGCAACC
56	RtcR-XhoI	ACTCTCGAGTTAATTCTGTAAAACGTCCCACGTCAGCCCAA AACG
57	pSRK Insert F	CCGGCTCGTATGTTGTGTGG
58	pSRKTc Rev	CAAGGCGATTAAGTTGGGTAACG
59	pNN387RtcRNt-NotI-F	ATCACTGCGGCCGCTTACGTCTCCTCCCGCTCCTG
60	pNN387-Prsr-HindIII-R	ATCACTAAGCTTTGCCATGGTTATTGCAACCAGCGT
61	NheI-STM14_3218-3220-F	ATCACTGCTAGCGCAAGGAATAATTATGCC
62	HindIII-STM14_3218-3220-R	ATCACTAAGCTTCCGGTTAAATTAGAGGTGTTAG
63	STM3521_upstream-L	TCTAGACGCTGGCGATATCCTTCTTTAATCC
64	STM3521_upstream-R	GGATCCGTTATTCTCCATGCCATGG
**Sequencing of Tn5 Insertion Barcodes**		
65	Right_Reverse	GTCTCTTATACACATCTCAACCCTG
66	Right_Forward_983	AATTGGTTGTAACACTGGCAGAGCATTA
67	2nd_PCR_Tn5_EZ_Right_ Forward	AATGATACGGCGACCACCGAGGCCATTAATACGACTCACTA TAGGGAGACCGGCC
68	Tn5_8_Base_Index (Series)	CAAGCAGAAGACGGCATACGAGAT-8-base-index- CCCGTCCCACCGTCTCTTATACACATCTCAACC
69	Tn5_EZ_Right_Seq	GGCCATTAATACGACTCACTATAGGGAGACC GGCCT
70	Tn5_EZ_Index_Seq	GGTTGAGATGTGTATA AGAGACGGTGGGACGGG
**Reporter Strain Construction for Deletion Mutants Based on Tn5 Insertion Screen**		
71	ckpcnB-F	GCAGGAGATGTCTGTTGAGGAGTTG
72	ckpcnB-R	ACCAAGATGGGTGAGATGCTGATG

73	ckhupA-F	CGACTGCGAAGAACGTGATATTGAACTG
74	ckhupA-R	GCGAATATGTTCTGGCGGTTTGCTG
75	ckfis-F	GTTCAACGGAGAAGCTGAATACGACAG
76	ckfis-R	GGAACTATCATAGGACCAGACGATACG
77	ckhflX-F	GTTGAAGAACACGGTCAGCCAGATGG
78	ckhflX-R	GCTACCGCCTCAATACGACCTGAATGAC
79	ckrcsD-F	TTGTACCGTTCTGCGAAGGTTGTAC
80	ckrcsD-R	GTTCAAGTGATTTGCGAATACCGAACAG
81	ckhupB-F	GAACCGTCTGGAATGCAGGTTGTAAC
82	ckhupB-R	GTTATTGCTTCCGCCAATCAGGTAACC
83	ckrpoS-F	CGCCCATAATGATACGATGCT
84	ckrpoS-R	GCGAACACTATCCACAAGCGT
85	ckppk-F	TATGTCATCGGACAGGACTGCGTCTG
86	ckppk-R	CTGACGTAACGTATGGGTGCCTACG
87	ckompC-F	TCGTATTTGTACGCCGGAATAAGG
88	ckompC-R	TGCGGAGAATGGACTTGCC
**Gifsy-1 Gene Deletions Construction**		
89	3211-Red-F	ATGAAATACTCTAGAGTTGAACAGTCAACAGGTGTGTAGGC TGGAGCTGCTTC
90	3211-Red-R	CTAGGAGGGCTTAAACGCCATTTTTGTGCTCCATATGAATAT CCTCCTTA
91	3218-Red-F	ATGCCAGCATCGGTAATTAGCTTTATTAATATGTGTGTAGGC TGGAGCTGCTTC
92	3220-Red-R	TTAGTTCAGCGCTTTAAATTTGGTGTATAAACCATATGAATA TCCTCCTTA
93	3177-Red-F	ATGGACCAGATAGCGAACCTGGTCATTGATTTTGTGTAGGC TGGAGCTGCTTC
94	3177-Red-R	TTATCGCCCTCCTCCAGCAATACCCCCGTCACGCATATGAAT ATCCTCCTTA
95	Ck-D3211-F	AAGATGGTGAGCCTCGCTTGC
96	Ck-D3211-R	ATTCGGCTGGCAAAGCTCTGG

97	Ck-D3218-20-F	AAGAGGTCGACGAGAGGGACG
98	Ck-D3218-20-R	ACGGCTACTTCGTGGGCG
**Additional plasmid construction**		
99	NdeI-3218-20-F	ATCAGTCATATGAGCAAGGAATAATTATGCC
100	XhoI-3218-R	ATCACTCTCGAGTTATCCATTTTCACTCACC
101	Xho-I-3218-20-R	ATCACTCTCGAGTTAAATTAGAGGTGTTAGTTCAGC
102	NdeI-3219-20-F	ATCACTCATATGCGATAGGTCTTTTTGTGACC

Supplemental Figures-

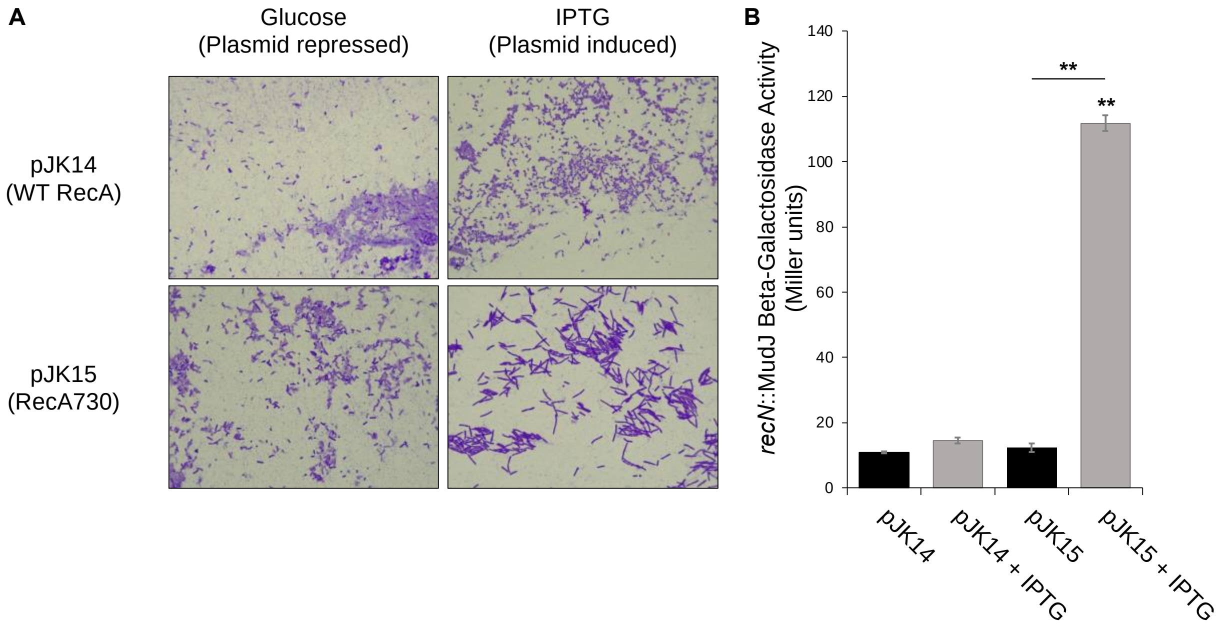

Figure S1. The *S.* Typhimurium RecA730 variant induces the SOS response in the absence of DNA damage. (A) Crystal violet staining and bright field microscopy (1000x magnification) of *S.* Typhimurium 14028s WT cells carrying pJK14 (expresses WT *recA* from *lacp*) or pJK15 (expresses *recA730* from *lacp*), grown in the presence of 0.2% glucose to repress expression from the *lacp* or 1 mM IPTG to induce expression (90 min). (B) Beta-galactosidase assay for strain TT23994 (*S.* Typhimurium LT2 *recN557*::MudJ) which expresses LacZ upon induction of the SOS response (5). Cells carried pJK14 or pJK15 and were assayed after growth without inducing agent or with the addition of 1 mM IPTG (90 min). Data shown is representative of at least 2 biological replicates; error bars represent ±1 standard deviation. Significant differences in beta-galactosidase activity between uninduced and induced samples are indicated by asterisks above horizontal bars centered between the compared data sets; significance differences in beta-galactosidase activity for the RecA730 expression strain (uninduced or induced) in pairwise comparison to the RecA expression strain (uninduced or induced) are indicated by asterisks above the RecA730 samples (**, *P* value < 0.01).

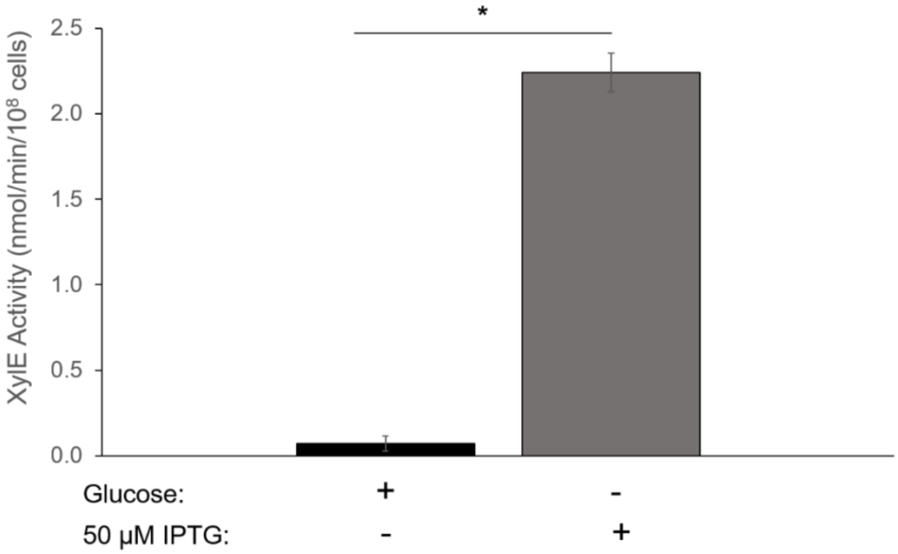

Figure S2. RecA is not required for a constitutively active RtcR variant to stimulate RpoN-dependent transcription from *rsrp*. A XylE assay was performed to assess transcription from *rsrp* in the Δ*recA*::*kan* Δ*rsr*::*xylE* reporter strain (JEK26) carrying pCH6 which expresses a constitutively active variant of RtcR ($RtcR_{con}$). $RtcR_{con}$ lacks the regulatory domain and is not dependent on sensing a signal for activation (1). Cells were assayed after growth in the presence of 0.2% glucose to repress expression of the plasmid or 50 µM IPTG to induce expression (90 min). Data shown is representative of 3 biological replicates, each with two technical replicates; error bars represent ±1 standard deviation. Statistical significance was determined by Student's *t* test (*P < 0.001).

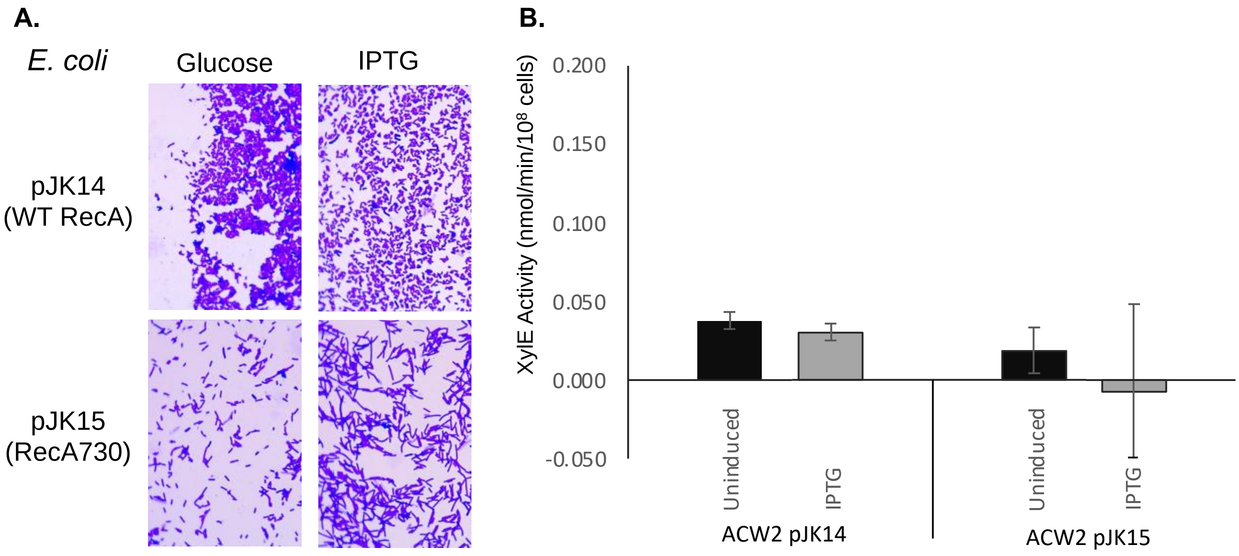

Figure S3. RtcR is not activated to stimulate RpoN-dependent transcription of the *E. coli rtcBA* operon during the SOS response. (A) Crystal violet staining and bright field microscopy (1000x magnification) of *E. coli* cells carrying plasmids that express *S.* Typhimurium WT RecA (pJK14) or RecA730 (pJK15), grown in the presence of 0.2% glucose to repress expression from *lacp* or 1 mM IPTG to induce expression (90 min). (B) XylE activity assays were performed to assess activation of the *rtcBp* promoter in the *E. coli* reporter strain (ACW2) carrying pJK14 or pJK15. Mid-log phase cultures were split and one of each pair had 1 mM IPTG added to induce RecA or RecA730 expression from pJK14 or pJK15, respectively, while the other was uninduced. After 90 min growth the cultures were assayed for XylE activity. ACW2 carrying pCH6, which expresses the constitutively-active $RtcR_{con}$, was utilized as a positive control; XylE activity following treatment with 50 μM IPTG for 90 min was 8.0 ± 0.3 nmol/min/10^8 cells (234-fold higher than uninduced); data not included in bar graph. Data shown is representative of 3 biological replicates, each with two technical replicates; error bars represent ±1 standard deviation. Statistical significance for induced versus uninduced, as determined by Student's *t* test, was $P > 0.05$ (not significant).

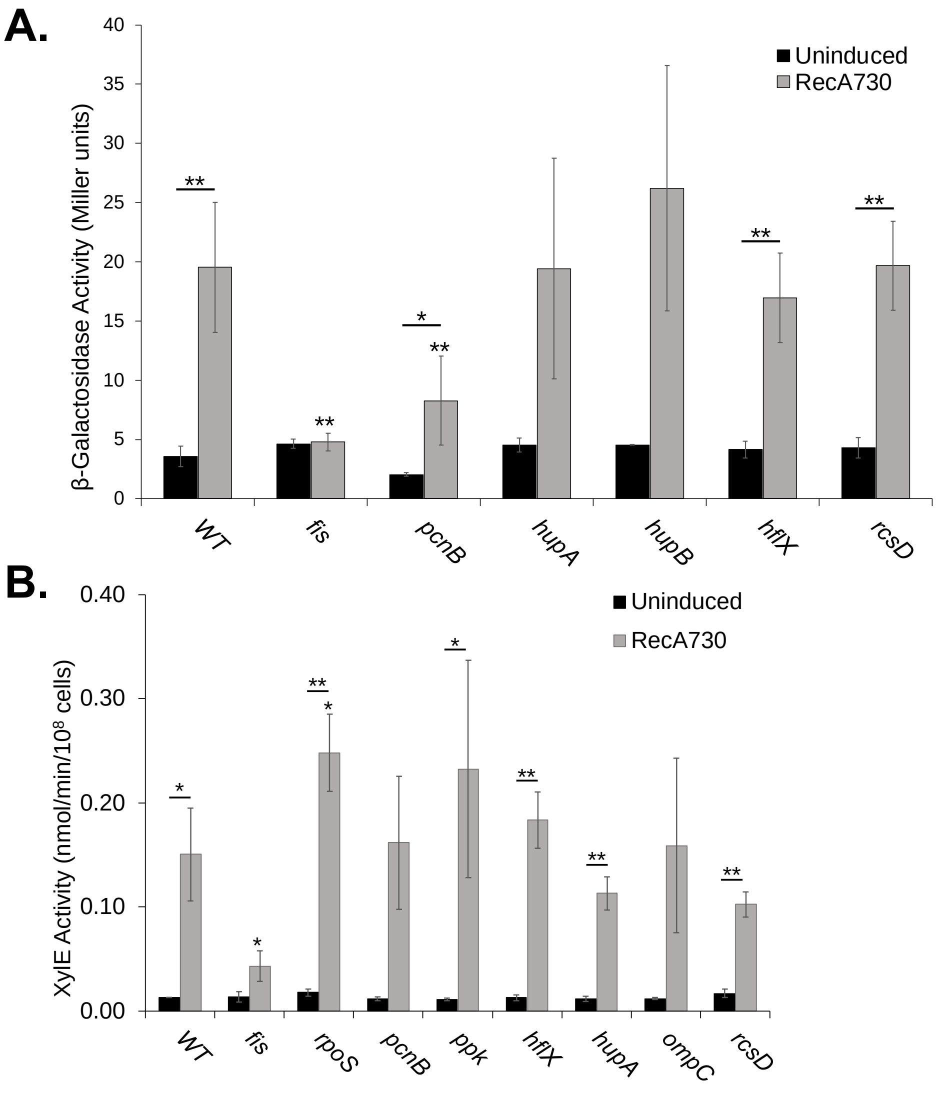

Figure S4. Reporter assays to assess activation of transcription from *rsrp* in deletion mutants for genes that were identified in the Tn5 mutagenesis screen. (A) Beta-galactosidase assays were conducted for single gene deletion mutants co-maintaining pJK15 (RecA730 expression vector) and pJK19 (*rsrp-lacZ* reporter); the indicated gene (on x-axis) was replaced with a *kan* resistance marker. (B) XylE activity assays were conducted on Δ*rsr*::*xylE* reporter strains with select single gene deletion mutants (genes indicated on x-axis) and carrying pJK15. For (A) and (B), cultures were split at mid-log and assayed after 90 min growth without inducing agent or with 1 mM IPTG. All data shown is representative of at least 3 biological replicates, each with two technical replicates; error bars represent ±1 standard deviation. Significant differences in XylE or beta-galactosidase activity between uninduced and induced (RecA730 expression) samples are indicated by asterisks above horizontal bars centered between the paired samples; significance differences in XylE or beta-galactosidase activity for mutant strains and treatments (uninduced or induced) in comparison to the WT counterpart are indicated by asterisks above the mutant samples (*, *P* value < 0.05; **, *P* value < 0.01).

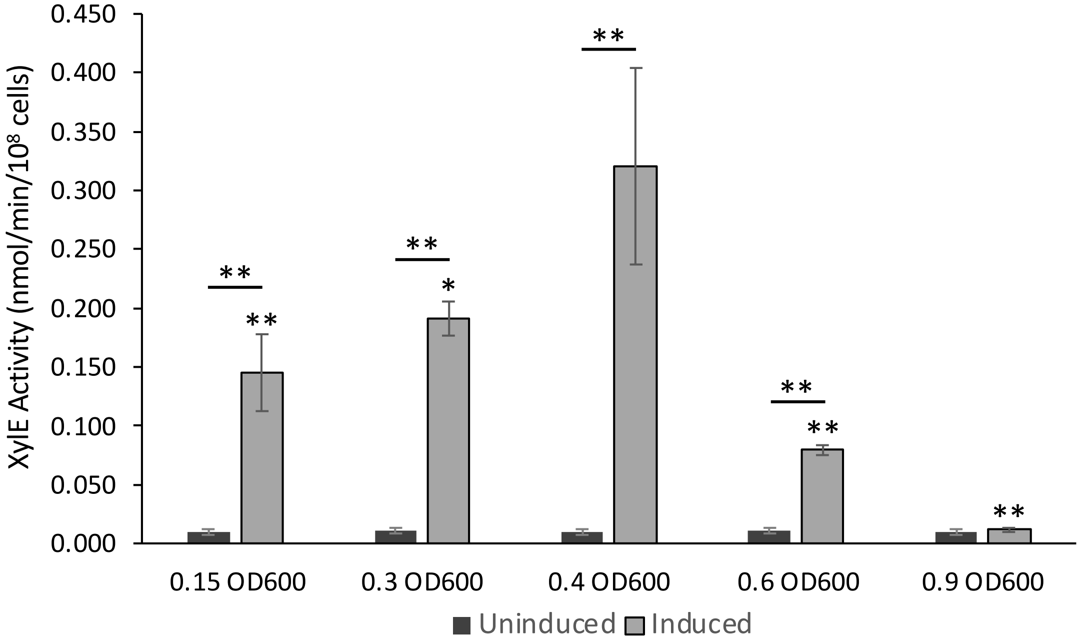

Figure S5. RtcR activation shows a dependence on growth phase. XylE assays were performed with the 14028s WT Δ*rsr*::*xylE* reporter strain containing pJK15 (RecA730 expression vector). Overnight cultures for each biological replicate were subcultured 1:100 in fresh LB-Tet and aliquots were taken for induction with 1 mM IPTG at the transition from lag to logarithmic growth (0.15 OD_{600}), early- to mid-log phase (0.3 and 0.4 OD_{600}), and late-log phase (0.6 and 0.9 OD_{600}). The uninduced samples were taken at 0.4 OD_{600}. Induced and uninduced samples were grown for an additional 90 min and cells were harvested to determine XylE activity. Activity increased for samples induced for RecA730 expression from late lag to early- to mid-log phases and dramatically decreased for samples induced during late-log growth. Data shown is for at least 3 biological replicates, each with two technical replicates; error bars represent ±1 standard deviation. Significant differences in XylE activity between uninduced and induced samples are indicated by asterisks above the horizontal bar centered between the paired samples; significance differences in XylE activity for induced samples in comparison to the peak activity (0.4 OD_{600} samples) is indicated by asterisks above the samples (*, P value < 0.05, **, P value < 0.01).

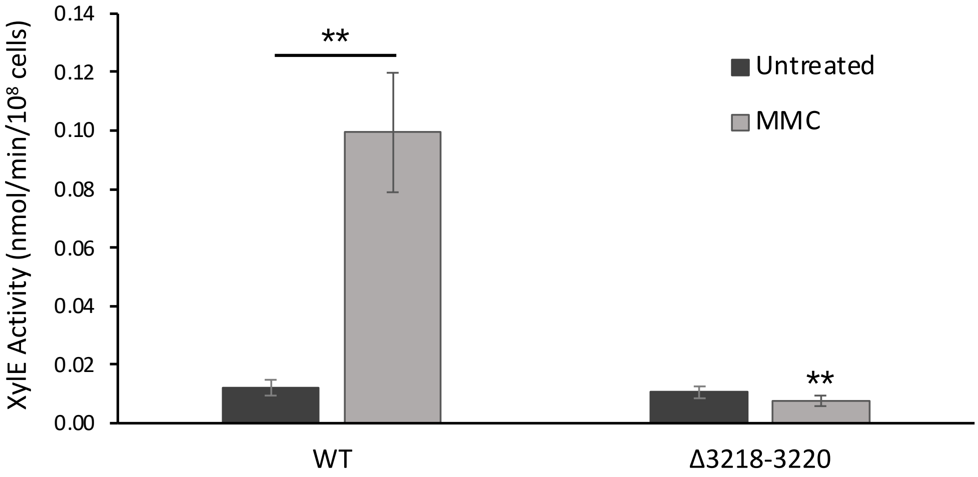

Figure S6. *S*. Typhimurium 14028s deleted for the Gifsy-1 STM14_3218-3220 region is defective for activation of transcription from *rsrp* during MMC-induced SOS response. XylE assays were performed with the 14028s WT and ΔSTM14_3218-3220 Δ*rsr*::*xylE* reporter strains, which contain no plasmids. Cultures were split at mid-log phase and one of each pair was treated with 3 μM MMC to initiate the SOS response for RtcR activation conditions; cells were assayed after 90 min growth. Data shown is for 4 biological replicates, each with two technical replicates; error bars represent ±1 standard deviation. Significant differences in XylE activity between uninduced and induced samples are indicated by asterisks above the horizontal bar centered between the paired samples; significance differences in XylE activity for the mutant strain and treatments (uninduced or induced) in comparison to the 14028s WT counterpart is indicated by asterisks above the mutant samples (*, P value < 0.05, **, P value < 0.01).

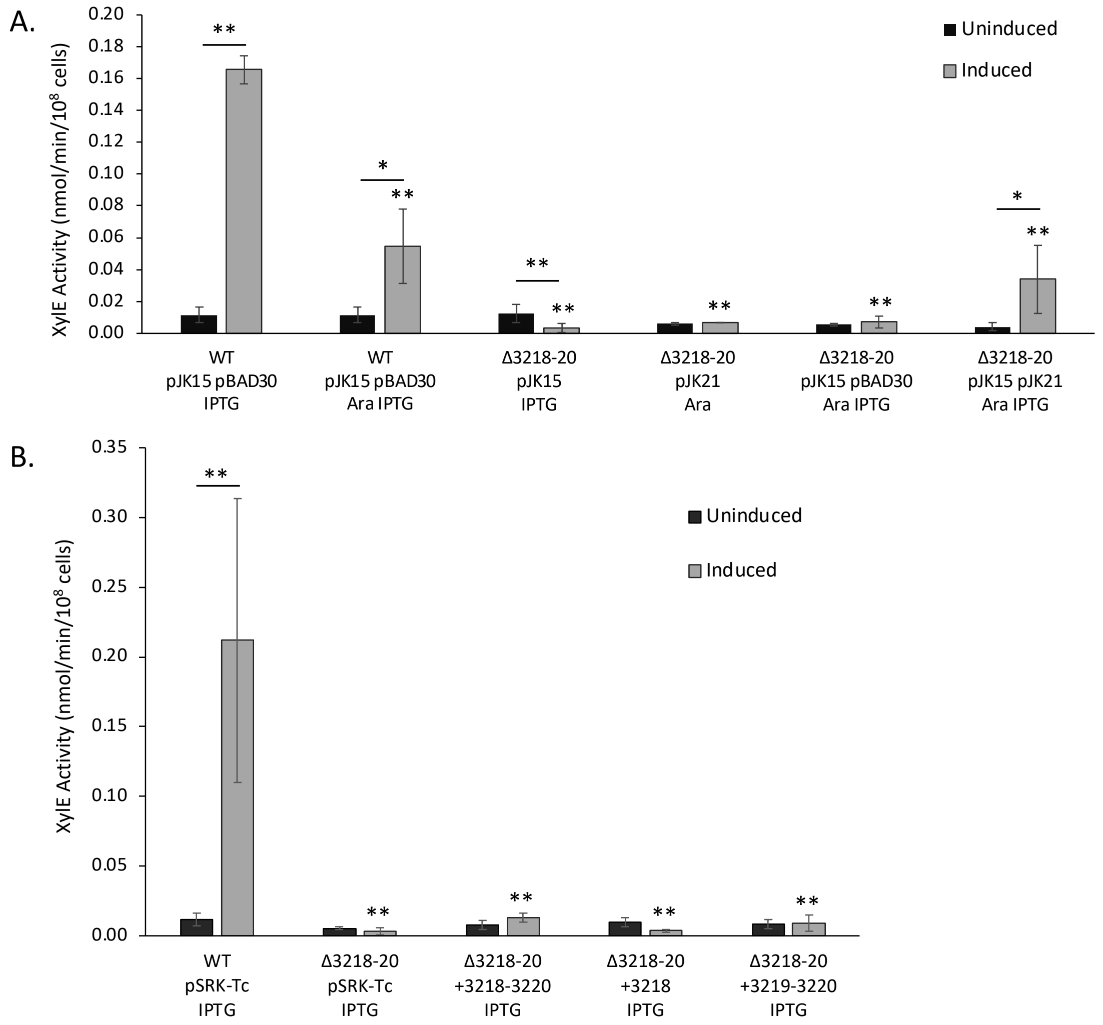

Figure S7. Assays for complementation of *S*. Typhimurium 14028s ΔSTM14_3218-3220 for RtcR activation by heterologous expression of STM14_3218-3220. For the XylE activity assays in both (A) and (B), cultures of reporter strains were grown to OD_{600} = 0.4, split for uninduced and induced samples, and cells were assayed for XylE activity 90 min post-induction. (A) XylE activity assays were performed with 14028s wild-type (WT) and ΔSTM14_3218-3220 (Δ3218-20) Δ*rsr*::*xylE* reporter strains in the presence and absence of STM14_3218-3220 expressed from *araBADp* on pBAD30 (pJK21) and RecA730 expressed from *lacp* on pSRK-Tc (pJK15). Expression from *araBADp* was induced with 0.05% arabinose (Ara) and expression from *lacp* was induced with 1 mM IPTG (IPTG) (inducers for each strain are indicated on the x-axis). The empty expression vector (pBAD30) was included in control strains to assess any effect of the vector on XylE activity. (B) XylE activity assays were performed with 14028s WT and ΔSTM14_3218-20 Δ*rsr*::*xylE* reporter strains containing the empty pSRK-Tc vector or pSRK-Tc with STM14_3218-3220 (+3218-20), STM14_3218 (+3218), or STM14_3219-3220 (+3219-20) under *lacp* control (pAK102, pAK101, and pAK103, respectively; **Table S3**). Expression from *lacp* was induced with 1 mM IPTG. The RecA730 expression vector cannot be co-maintained with these pSRK-Tc-based plasmids, so treatment with 3 µM MMC was utilized to induce RtcR activation conditions. Data shown is for at least 3 biological replicates, each with two technical replicates; error bars represent ±1 standard deviation. Significant differences in XylE activity between uninduced and induced samples are indicated by asterisks above the horizontal bar centered between the paired samples; significant differences in XylE activity in comparison to the 14028s WT counterpart (first condition in (A) and (B)) is indicated by asterisks above the samples (*, *P* value < 0.05, **, *P* value < 0.01).

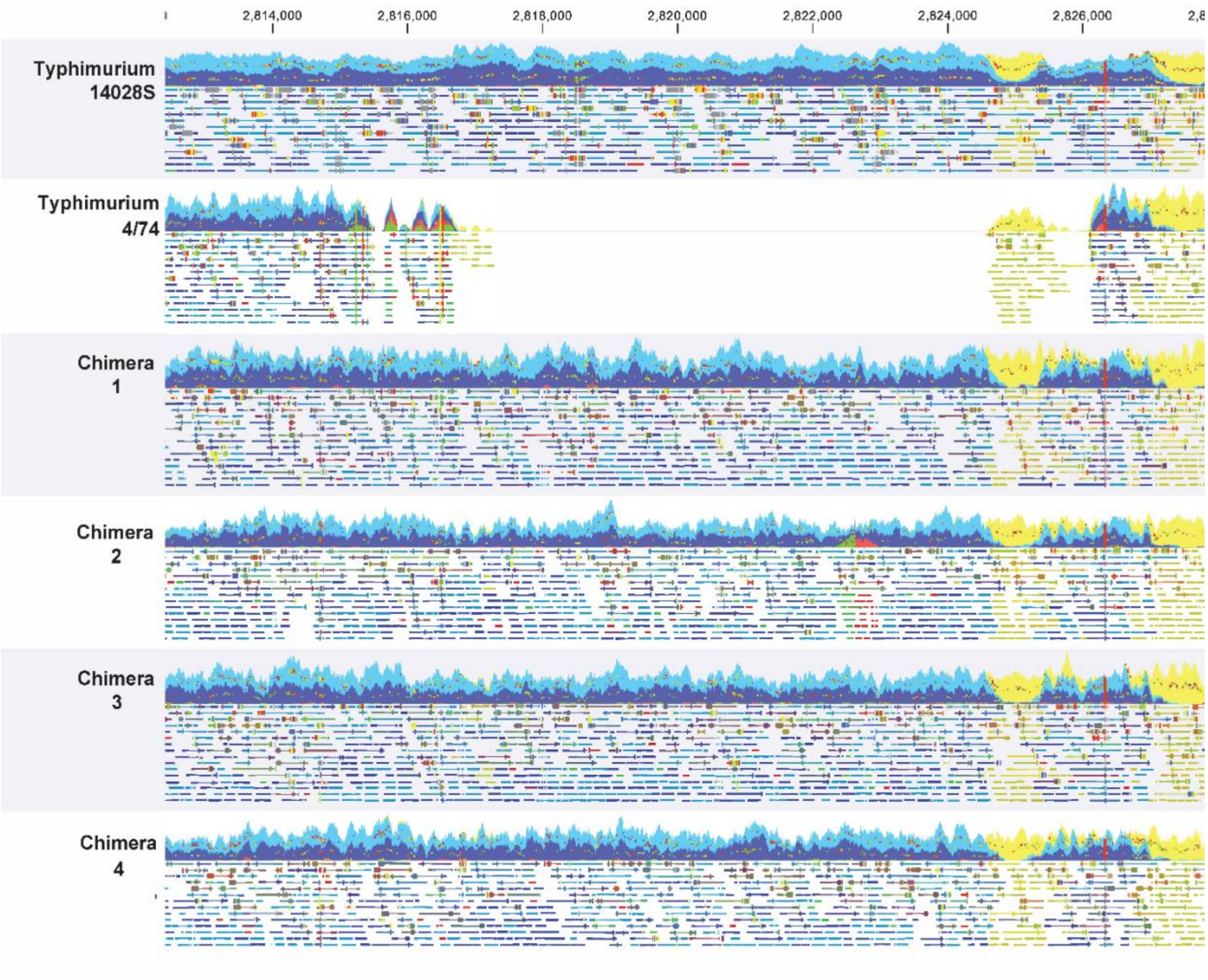

Figure S8. Comparison of *S*. Typhimurium strain 14028s genomic sequence gained by lateral transfer and *S*. Typhimurium 4/74 genomic sequence replaced in 4/74-14028s chimeras that acquired genetic elements required for RtcR-activation. CLCBio (qiagenbioinformatics.com) was utilized to generate of the image shown here comparing mapped reads for the transduced region in the four chimeric strains with the sequence for the donor strain 14028s and the recipient strain 4/74. The genome positions for 14028s are marked at the top of the image. The color code for the mapped reads is: single-end reads mapped in forward and reverse directions are green and red, respectively; paired-end reads mapped in forward and reverse directions are dark blue and light blue, respectively; and reads mapped in multiple places are yellow. The rest of each chimeric genome has the indels and SNPs associated with 4/74 with no other 14028S genome region in common (data not shown).

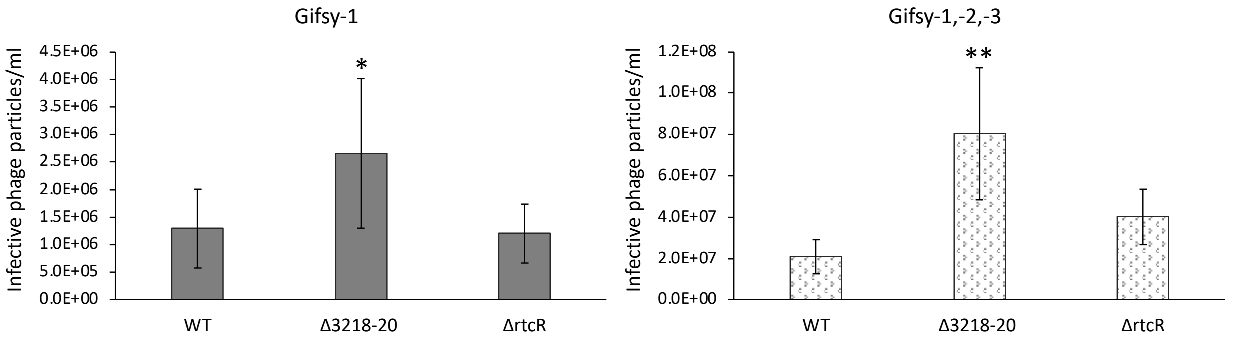

Figure S9. Gifsy phage production during the SOS response in *S*. Typhimurium 14028s WT, ΔSTM14_3218-3220, and Δ*rtcR* strains. Mid-log phase cultures (OD_{600} = 0.4) of *S*. Typhimurium 14028s WT (MA5958), ΔSTM14_3218-3220 (JEK132), and Δ*rtcR* (DJS101) in LB with 10 mM $MgSO_4$ were split for uninduced and induced (treated with 2 µg/ml MMC) samples and grown for 4 hours. Cultures were centrifuged and infective phage particles in the supernatants were quantitated by spotting 10-fold serial dilutions on EBU plates (3) spread with mid-log cultures of 14028s Gifsy-1 [-] (MA5973) to detect Gifsy-1 phage or 14028s Gifsy-1[-] Gifsy-2[-] Gifsy-3[-] (MA6052) to detect all three Gifsy phages; phage infection foci appear as dark blue-green specks on a light green background, as described in (6). The supernatants from uninduced samples for WT, ΔSTM14_3218-3220, and Δ*rtcR* strains had equivalent infective phage particle concentrations for Gifsy-1 (8.7E+03, 6.4E+03, and 5.5E+03, respectively) and for all 3 Gifsy phages (1.4E+05, 1.6E+05, and 1.4E+05, respectively). The infective phage particle concentrations for supernatants from MMC-induced cultures are shown in the bar graphs. Data shown is for 6 biological replicates, each with two technical replicates; error bars represent ±1 standard deviation. Significant differences for phage titer in comparison to the WT is indicated by asterisks above the mutant samples (*, P value < 0.05, **, P value < 0.01).

References:

1. Kurasz JE, Hartman, C. E., Samuels, D. J., Mohanty, B. K., Deleveaux, A., Mrázek, J., and Karls, A. C. 2018. Genotoxic, metabolic, and oxidative stresses regulate the RNA repair operon of *Salmonella enterica* serovar Typhimurium. J Bacteriol 200:e00476-18.
2. Datsenko KA, and W, Banner. L. 2000. One-step inactivation of chromosomal genes in *Escherichia coli* K-12 using PCR products. PNAS 97:6640-6645.
3. Maloy SR. 1990. Experimental techniques in bacterial genetics. Jones and Bartlett, Boston.
4. Porwollik S, Santiviago, C. A., Cheng, P., Long, F., Desai, P., Fredlund, J., Srikumar, S., Silva, C. A., Chu, W., Chen, X., Canals, R., Reynolds, M. M., Bogomolnaya, L, Shields, C., Cui, P., Guo, J., Zheng, Y., Endicott-Yazdani, T., Yang, H.-J., Maple, A., Ragoza, Y., Blondel, C. J., Valanzuela, C., Andrews-Polymenis, H., and McClelland, M. 2014. Defined single-gene and multi-gene deletion mutant collections in *Salmonella enterica* sv Typhimurium. PLoS One 9:e99820.
5. Bunny K, Liu, J., and Roth, J. 2002. Phenotypes of *lexA* mutations in *Salmonella enterica*: evidence for a lethal *lexA* null phenotype due to the Fels-2 prophage. J Bacteriol 184:6235-6249.
6. Figueroa-Bossi N, and Bossi, L. 1999. Inducible prophages contribute to *Salmonella* virulence in mice. Mol Microbiol 33:167-176.
7. Figueroa-Bossi N, Uzzau, S., Maloriol, D., and Bossi, L. 2001. Variable assortment of prophages provides a transferable repertoire of pathogenic determinants in *Salmonella*. Mol Microbiol 39:260-272.
8. Cherepanov PP, and Wackernagel, W. 1995. Gene disruption in *Escerichia coli*: TcR and KmR cassettes with the option of Flp-catalyzed excision of the antibiotic-resistance determinant. Gene 158:9-14.
9. Khan SK, Jennifer Gaines, R. Martin Roop II, and Stephen K. Farrand. 2008. Broad-host-range expression vectors with tightly regulated promoters and their use to examine the influence of TraR and TraM expression on Ti plasmid quorum sensing. Appl Environ Microbiol 74:10.
10. Horton RM, Cai, Z., Ho, S. N., and Pease, L. R. 1990. Gene splicing by overlap extension: tailor-made genes using the polymerase chain reaction. BioTechniques 8:528-535.
11. Khan SK, Gaines, J., Roop II, R. M., and Farrand, S. K. 2008. Broad-host-range expression vectors with tightly regulated promoters and their use to examine the influence of TraR and TraM expression on Ti plasmid quorum sensing. Appl Environ Microbiol 74:5053-5062.
12. Perkins-Balding D, Duval-Valentine, G., and Glasgow, A. C. 1999. Excision of IS*492* requires flanking target sequences and results in circle formation in *Pseudoalteromonas atlantica*. J Bacteriol 181:4937-4948.
13. Guzman LM, Belin, D., Carson, M. J., and Beckwith, J. 1995. Tight regulation, modulation, and high-level expressiom by vectors containing the arabinose PBAD promoter. J Bacteriol 177:4121-4130.
14. Grana D, Youderian, P., and Susskind, M. M. 1985. Mutations that improve the *ant* promoter of *Salmonella* prophage P22. Genetics 110.
15. Jones PW, Collins P, Aitken MM. 1988. Passive protection of calves against experimental infection with Salmonella typhimurium. Vet Rec 123:536-41.
16. McKinney J, Guerrier-Takada, C., Galán, J., and Altman, S. 2002. Tightly regulated gene expression system in *Salmonella enterica* serovar Typhimurium. J Bacteriol 184:6056-6059.
17. de Moraes MH, Desai, P., Porwollik, S., Canals, R., Perez, D. R., Chu, W., McClelland, M., and Teplitski, M. 2017. *Salmonella* persistence in tomatoes requires a distinct set of metabolic functions identified by transposon insertion sequencing. Appl Environ Microbiol 83:e03028-16.
